# A Review of Protein- and Peptide-Based Chemical Conjugates: Past, Present, and Future

**DOI:** 10.3390/pharmaceutics15020600

**Published:** 2023-02-10

**Authors:** Emily Holz, Martine Darwish, Devin B. Tesar, Whitney Shatz-Binder

**Affiliations:** 1 Department of Pharmaceutical Development, Genentech, Inc., 1 DNA Way, South San Francisco, CA 94080, USA; 2 Department of Protein Chemistry, Genentech, Inc., 1 DNA Way, South San Francisco, CA 94080, USA

**Keywords:** bioconjugation, protein therapeutics, protein–polymer conjugates, protein delivery, ADC, AOC, protein conjugate vaccines, antibody delivery

## Abstract

Over the past few decades, the complexity of molecular entities being advanced for therapeutic purposes has continued to evolve. A main propellent fueling innovation is the perpetual mandate within the pharmaceutical industry to meet the needs of novel disease areas and/or delivery challenges. As new mechanisms of action are uncovered, and as our understanding of existing mechanisms grows, the properties that are required and/or leveraged to enable therapeutic development continue to expand. One rapidly evolving area of interest is that of chemically enhanced peptide and protein therapeutics. While a variety of conjugate molecules such as antibody–drug conjugates, peptide/protein–PEG conjugates, and protein conjugate vaccines are already well established, others, such as antibody–oligonucleotide conjugates and peptide/protein conjugates using non-PEG polymers, are newer to clinical development. This review will evaluate the current development landscape of protein-based chemical conjugates with special attention to considerations such as modulation of pharmacokinetics, safety/tolerability, and entry into difficult to access targets, as well as bioavailability. Furthermore, for the purpose of this review, the types of molecules discussed are divided into two categories: (1) therapeutics that are enhanced by protein or peptide bioconjugation, and (2) protein and peptide therapeutics that require chemical modifications. Overall, the breadth of novel peptide- or protein-based therapeutics moving through the pipeline each year supports a path forward for the pursuit of even more complex therapeutic strategies.

## 1. Introduction

Proteins and peptides have played a foundational role in the treatment of diseases for nearly a century, beginning with the first commercial use of insulin in 1923. Early protein-based therapeutics, developed prior to the introduction of recombinant DNA technology, were limited by their immunogenicity. The use of chemical conjugation to enhance the properties of proteins dates back to at least the 1970s, when Frank Davis hypothesized that conjugation of a hydrophilic polymer such as PEG could reduce the immunogenicity of non-native proteins and unexpectedly discovered that PEGylation improved the circulating half-life of proteins as well [1,2,3,4]. Since then, at least 30 polymer–protein and polymer–peptide conjugates have been approved by the FDA [5], each of which use polymer conjugation to improve their pharmacokinetics and shield them from antidrug antibody recognition. In addition, the 50-year span of research into the properties of polymer conjugates has unveiled many new properties in this class of therapeutics that have expanded both their prevalence and mechanisms of action in the clinical space.

While the protein represents the active pharmaceutical ingredient (API) for polymer conjugates, other chemically enhanced therapeutics take the opposite approach, using conjugation to proteins as the delivery strategy. Inspired by Paul Ehrlich’s “magic bullet” approach, conceptualized in 1907, antibody–drug conjugates (ADC) were developed to target malignant cells for the delivery of cytotoxic drugs. In this approach, the role of the antibody is to improve circulating half-life and cell-specific uptake of the drug in its target tissue, thereby improving its therapeutic index (TI). Since the first report in 1958 [6], 12 ADCs have been approved to date, while over 80 are currently undergoing clinical trials. Another example of using proteins for a purpose other than as API is in the case of protein-based vaccines, with the first approved hepatitis B vaccine some 30 years ago [7]. In this case, the protein is acting as an agonist to stimulate an immune response, thereby increasing the efficacy of the vaccine. Since then, various novel protein-based approaches have been developed to offer a wider variety of tools for effective vaccines against difficult pathogens. More recently, antibody conjugates have expanded beyond cytotoxic drugs to include novel payloads such as oligonucleotides, offering new and innovative ways to tackle what were previously thought to be undruggable targets. For example, the recent advancements of oligonucleotide-based therapies, designed to bind noncoding RNAs and toxic RNAs associated with disease pathogenesis, have greatly expanded the numbers and types of selectable targets.

The advancement of these novel modalities has nonetheless brought with it new challenges. For polymer–protein and polymer–peptide conjugates, recent progress has been directed towards the design of novel polymers and conjugation chemistries to retain or improve biological activity and evade immune recognition in vivo. Meanwhile, the refinement of both the payload and linker chemistries to improve safety and on-target delivery has been a major focus for ADCs. In the case of therapeutic oligonucleotides, the druggable space within the genome is quite expansive, and their efficacy is mostly limited by ineffective delivery to their intracellular targets. Thus, devising and evaluating an array of delivery strategies has become a critical component of the development of this emerging class of therapeutics. Likewise, the field of protein conjugate vaccines has also benefited from recent innovations leveraging modern protein engineering and chemical conjugation strategies to address the long-standing challenge of generating a robust immune response to difficult carbohydrate antigens.

In this review, we will highlight the diverse role proteins play in the development of therapeutics by examining the recent developments in four major fields which use protein conjugation as a key component of the therapeutic design: antibody–drug conjugates, protein/peptide–oligonucleotide conjugates, protein conjugate vaccines, and polymer–protein/peptide conjugates. The breadth of molecules that will be covered spans from approved products all the way to newer endeavors, with in vitro or limited data only.

## 2. Therapeutics That are Enhanced by Protein Conjugation

### 2.1. Antibody–Drug Conjugates

Monoclonal antibodies (mAbs) that are specific for cell-surface antigens overexpressed on cancer cells, yet have limited or no therapeutic activity on their own, can be armed through chemical conjugation of potent cytotoxic compounds. Upon target-binding and internalization of ADCs, their small molecule payloads can be released through a variety of mechanisms, resulting in targeted cancer cell death, ideally with low systemic toxicity. Successful ADCs must be stable in circulation, highly specific for malignant cells, and able to efficiently release active cytotoxics after delivery to these cells. This “magic bullet” approach has shown recent clinical success, with 12 therapeutics approved by the FDA since 2000 (Table 1). Despite these approvals, we are still early in our understanding of these complex therapeutics, with clinical success using this platform dependent on all components of the molecule being optimized for maximum specificity, efficacy, and tolerability.

**Figure 1:**
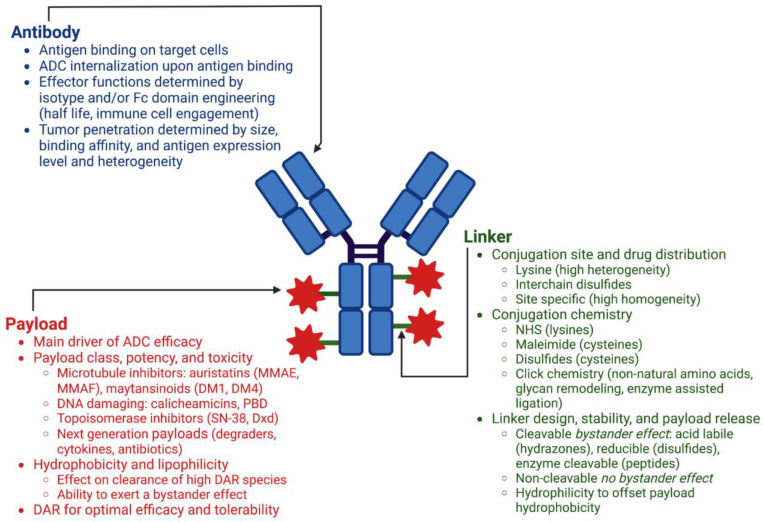
ADCs are made up of the antibody (blue), the payload (red), and the chemical conjugation (green) that brings them together. Functions and the most critical design considerations for each component have been listed. Created with BioRender.com.

The three main components of an antibody–drug conjugate are the antibody, the small molecule payload, and the chemical linker connecting the two (Figure 1). There are numerous considerations for each of these components during ADC design, and the modular nature of this platform allows for various combinations of each to be constructed and tested in in vitro and in vivo models of efficacy, toxicity, and PK, enabling optimization of the final therapeutic candidate.

#### 2.1.1. Antibody

ADC efficacy and safety is dependent on the selection of an antibody that targets an antigen that is highly expressed on tumor cells, with little to no expression on nonmalignant cells. This specificity, or lack thereof, is one of the main determinants of toxicity and clinical success, as any cells displaying the target will receive ADC [8]. In addition to high selectivity for the antigen of interest, sufficient amounts of drug to induce cell death must be able to be delivered, requiring high expression levels of the target antigen. This threshold expression level will differ by tumor type and ADC construct [9]. Upon binding of the ADC, antigen targets should be well internalized as activity of the ADC is dependent on payload release in response to conditions in the endosomal pathway. Homogeneity of antigen expression in the target tumor type is desired to deliver payload to all malignant cells; however, heterogeneity of antigen expression can be overcome by achieving a bystander effect through linker-drug design [10]. Established targets for approved ADCs include HER2, TROP2, and Nectin-4 for solid tumors and CD33, CD30, CD22, and CD79b for hematological malignancies (Table 1). Hematologic cell antigens from lineage specific markers provide an optimal target for ADCs and account for 6 of the 11 targets of approved ADCs. The treatment of solid tumors with ADCs is more challenging, with antigen expression heterogeneity and difficulty of ADC tumor penetration presenting the main obstacles.

In addition to target selection, the choice of antibody isotype is an important factor in the therapeutic potential of the final ADC. Human IgG1 antibodies are the dominant isotype used in approved ADCs, as well as those under development, with 11 approved ADCs employing IgG1 and two utilizing IgG4. IgG1 antibodies owe their popularity to their long serum half-lives, effector functions, ease of production, and conjugatability [11]. Human IgG1 antibodies consist of two heavy chains and two light chains joined together by four interchain disulfides: two connecting the heavy and light chains of the two Fab regions, and two connecting the heavy chains at the hinge region above the Fc. The structure of the Fc region imparts effector functions through binding to Fc receptors such as neonatal Fc receptor (FcRn) and Fc-γ receptor. These can include regulation of serum half-life through FcRn recycling and mediation of secondary immune functions such as complement-dependent cytotoxicity (CDC), antibody-dependent cell-mediated cytotoxicity (ADCC), and antibody-dependent cell-mediated phagocytosis (ADCP) [12]. ADCs maintain similar properties to their starting naked antibodies, including their antigen-binding affinities, effector functions, and serum half-lives. Consequently, ADCs utilizing antibodies that have antitumor activities on their own retain those functionalities as an ADC, enabling payload independent antitumor activity. Such is the case for trastuzumab emtansine (Kadcyla^®^) which utilizes trastuzumab (Herceptin^®^), a previously approved monoclonal antibody for the treatment of HER2+ breast cancer [13]. Trastuzumab’s actions are twofold: it binds to HER2, thereby preventing downstream signaling, and it mediates ADCC through its effector functions [14,15]. Trastuzumab emtansine retains these properties plus the enhanced tumor killing capabilities imparted by the cytotoxic payload DM1, and is effective in the treatment of patients that were previously treated with Trastuzumab in combination with a traditional chemotherapeutic, taxane [16].

While the antitumor enhancement of immune cell engagement through effector functions may be desirable for some ADCs, recent attention has been paid to modulating these interactions through Fc domain engineering of IgG1 antibodies [12]. Tuning these interactions through glycoengineering or via point mutations imparts advanced control over Fc receptor binding, with glycoengineering strategies designed to enhance Fc receptor binding as well as mutations designed to eliminate effector function all together [17]. The choice of effector enhanced or effectorless antibodies for ADC design will depend on disease specific factors and weighing the benefits of half-life extension and potential enhanced antitumor activity through immune cell engagement versus the potential negative effects on toxicity and tumor cell localization caused by nonspecific uptake by immune cells. The only approved ADC with enhanced Fc receptor binding, belantamab mafodotin, which is afucosylated, was voluntarily withdrawn in 2022 for not meeting the primary endpoint of its confirmatory Phase 3 trial. This ADC was plagued by severe ocular toxicities that required it to be available only through a risk evaluation and mitigation strategy program. While the exact causes of these toxicities require further investigation, ocular toxicities have been observed in previous clinical ADC candidates using MMAF and may also be attributed in part to the enhanced effector functions of its mAb resulting in nonspecific uptake in corneal cells [18].

#### 2.1.2. Payload

Approved ADCs exclusively employ potent cytotoxics that are not suitable for systemic delivery, and exert their cell-killing effects as microtubule inhibitors, DNA damaging agents, or DNA transcription inhibitors (Table 1). These payloads all have nanomolar or subnanomolar activity as free drugs and favor their toxicity for cells which are quickly dividing, a set of criteria that were both deemed requirements by the field to produce an efficacious ADC after early efforts employing traditional chemotherapeutics failed [19,20]. Early ADCs relying on conventional chemotherapy drugs, such as doxorubicin and methotrexate, did not show improved potency over the free drugs alone, requiring high doses for activity, diminishing the therapeutic window [21,22,23]. With as little as 0.1% of administered ADC actually reaching tumor cells, selection of a potent cytotoxic as cargo was deemed critical to achieving concentrations in the tumor cell high enough to induce cell death [24]. With this potency comes toxicity, which requires thoughtful molecule design to maximize the TI as nonspecific uptake of ADCs into healthy cells is a main driver of toxicity.

While conjugation enhances the activity of an antibody against a specific tumor cell target, it in turn enables delivery of promising small molecule compounds with poor drug-like properties that could likely never be developed as a single agent. Poor aqueous solubility can be overcome by conjugation to a macromolecule such as an antibody. Once released inside the tumor cell, these same properties, such as charge, lipophilicity, and overall cell permeability of the released drug, dictate whether a bystander effect will be observed, and different tumor types will benefit from different outcomes. A desired bystander effect may result in additional tumor cell killing of cells adjacent to the targeted cells, whereas an undesired bystander effect may result in drug uptake and death in healthy cells. Lipophilic payloads, such as the microtubule inhibitor monomethyl auristatin E (MMAE) and the DNA damaging agent pyrrolobenzodiazepine (PBD), can diffuse across membranes, entering target-negative cells adjacent to target-positive cells that received the ADC. Tumors with target antigen heterogeneity can benefit from this bystander effect as a high local concentration of the cytotoxic drug is achieved through targeted mediated delivery of the ADC to the tumor microenvironment, enhancing the therapeutic efficacy. Another microtubule inhibitor and family member to MMAE, monomethyl auristatin F (MMAF), contains a *C*-terminal carboxylic acid, limiting its membrane permeability and ability to exert a bystander effect [25]. Belantamab mafodotin (Blenrep^®^), the only approved ADC to use MMAF, was voluntarily withdrawn in 2022 after a Phase 3 confirmatory trial failed to show improved progression-free survival over the standard treatment in relapsed or refractory multiple myeloma (Table 1).

While the use of highly potent cytotoxic compounds has yielded clinical success, the recent use of less toxic topoisomerase inhibitors in trastuzumab deruxtecan (Enhertu^®^) and sacituzumab govitecan (Trodelvy^®^) is notable. Due to their slightly lower potencies, higher DAR conjugates are able to be delivered, increasing payload concentration at the tumor cell without increasing toxicity [13]. In recent clinical and research efforts, other, lower-potency payloads that target specific cellular proteins such as Toll-like receptor agonists, STING agonists, and Bcl-2 inhibitors are emerging as novel payloads [26]. Payloads that are selective for intracellular proteins such as proteolysis targeting chimerics (PROTACs) or other bivalent chemical protein degraders are also being explored [27]. The use of more targeted, lower-potency payloads which are selective for specific proteins, coupled with antibodies that are specific for cell-surface antigens, may provide an opportunity to accomplish a high degree of selectivity and efficacy in disease cells, while decreasing potential for on- and off-target toxicity in healthy cells.

#### 2.1.3. Linker Chemistry and Conjugation Methods

Various conjugation chemistries have been developed for small molecule drug attachment to antibodies, predominantly to recombinantly expressed human IgG1s. Early ADCs, such as gemtuzumab ozogamicin (Mylotarg^®^), ado-trastuzumab emtansine (Kadcyla^®^) and Inotuzumab ozogamicin (Besponsa^®^), used non-site-specific attachment strategies, specifically amine reactive succinimidyl esters (NHS esters), which can react with solvent exposed lysine side chains. This approach enables conjugation to antibodies without requiring engineering or disruption of their native structures. This strategy results in a heterogeneous mixture of antibody conjugated with varying amounts of drug, with the reported drug-to-antibody ratio (DAR) being an average of the different species. This method not only creates heterogeneity in the ADCs with regard to the number of drugs conjugated per antibody, but also with the location of those drugs, as a standard IgG1 contains approximately 20 solvent exposed lysines [28].

All other approved ADCs utilize interchain disulfides (four for an IgG1) for conjugation using thiol-reactive maleimide containing linkers. While not truly site-specific, conjugation to these cysteines results in a dramatic improvement in conjugate homogeneity over lysine conjugation strategies. A standard IgG1 antibody can accommodate up to eight conventional maleimide linker-drugs. While the calculated DAR is still an average of differently loaded species, the limited number of possible conjugation sites limits the drug load distribution.

Conjugation to the interchain disulfides of the antibody is accomplished after limited reduction with an excess of tris(2-carboxyethyl)phosphine or dithiothreitol. Maleimides can then react with these free thiols through a Michael addition forming a thiosuccinimide. This reaction is quick, specific, and can proceed at pH values as low as 5 and as high as 8, making it amenable to a wide range of antibody formulations [29]. Similar to lysine conjugation, conjugation to interchain disulfides does not require protein engineering to introduce reactive sites; however, it does disrupt interchain disulfide bonds in a heterogeneous manner [30]. Since sulfhydryls are liberated in pairs, antibodies conjugated through interchain disulfides typically have an even number of drugs conjugated with the reported DAR: an average of zero, two, four, six, and eight DAR species. Early studies suggested that a DAR between 3–4 was ideal for ADC efficacy and optimal PK [30]. In this DAR range, ADCs conjugated through interchain disulfides have a normal distribution of payload number, with small amounts of antibody conjugated with zero or eight drugs and most antibody species labeled with two or four drugs. This loading strategy limits the amount of inactive DAR0, while also limiting fully conjugated DAR8 species. ADCs with a DAR of eight have been shown to clear five times faster than lower DAR species, resulting in a decrease in tumor ADC exposure, an increase in off-target toxicity, and no commensurate increase in efficacy over lower DAR species [30]. The rapid clearance of DAR8 ADCs has been attributed to increased hydrophobicity of these conjugates due to their high drug load [31].

For the first 20 years of ADC approvals, all but one ADC had a DAR greater than this optimal range of 3–4 drugs per antibody (Table 1). In 2021, the approvals of trastuzumab deruxtecan (Enhertu^®^) and sacituzumab govitecan (Trodelvy^®^) challenged this accepted doctrine, with both ADCs having close to eight drugs per antibody. Both of these conjugates employ topoisomerase inhibitors as their cell-killing agent, which have a lower potency and lower toxicity than previously approved ADC payloads [13]. Despite the high DAR of sacituzumab govitecan, this ADC does not suffer from rapid clearance, and increased drug load correlates with improved in vivo efficacy [32]. The use of hydrophilic linkers in the design of these ADCs helps them accomplish their high DAR without negatively affecting PK [13].

Site-specific conjugation techniques have been developed to better control drug loading and to create homogeneous ADCs. The earliest and most notable platform is Genentech’s THIOMAB™ antibody technology, which uses engineered cysteines at specific sites in the antibody for uniform payload conjugation, leaving interchain disulfides intact. THIOMAB™ antibodies can be engineered to contain two, four, or six free cysteines for chemical conjugation [33]. The resulting THIOMAB™ antibody–drug conjugates have a high degree of homogeneity and improved TI over conventional ADCs [34]. Use of this technology requires thoughtful protein engineering and a high degree of sample processing to generate THIOMAB™ antibodies that are properly assembled, with free cysteines available for conjugation [29]. A number of ADCs using cysteine-engineered antibodies have entered the clinic for both solid tumor and hematological malignancies, but we have yet to see the therapeutic potential of this strategy realized in an approved ADC.

Other novel site-specific conjugation strategies have been developed for the production of homogeneous ADCs. A popular strategy that does not require protein reengineering of the antibody is disulfide rebridging. Disulfide rebridging uses bifunctional cysteine reactive linkers that attach to interchain disulfides, resulting in one drug attachment site per disulfide. Examples include Abzena’s ThioBridge™ and Sorrento’s C-Lock™, with the latter entering a Phase 1 clinical trial on a CD38 targeting antibody with a duostatin payload (DAR4) for treatment of relapsed or refractory systemic AL amyloidosis [35]. Other strategies include glycan remodeling, incorporation of unnatural amino acids for click chemistry, and enzyme-assisted modification. Glycan remodeling has been used by Mersana Therapeutics in their investigational ADC, XMT-1592, which is currently in Phase 1 clinical trials for the treatment of ovarian cancer and NSCLC. XMT-1592 consists of a Napi2B targeting antibody conjugated with Dolasynthen, a fleximer loaded with a proprietary microtubule inhibitor, using click chemistry after glycan remodeling of Asn297 [36]. The result is a site-specific ADC with a DAR of six that has shown an improved PK profile and payload accumulation at the tumor over a stochastic ADC with the same antibody and payload [36]. Despite significant advances in site-specific conjugation technologies, no approved ADCs to date employ these platforms. Many of the over 80 ADCs in clinical development do use site-specific conjugation methods to produce highly homogeneous therapeutics, and we look forward to the inevitable approval of ADCs utilizing these technologies.

With the use of cysteines as the predominant sites of payload attachment, maleimide–thiol conjugation was adopted early as a main conjugation chemistry by the field, but it was not until after years of development that the stability of this attachment was fully understood. Deconjugation through a retro-Michael reaction can occur in vivo, resulting in a DAR loss on the antibody and free linker-drug in circulation. The maleimide-linker drug can then exchange onto circulating free cysteines, such as that of albumin [37]. Premature release of payload in circulation through deconjugation results in decreased amounts of drug delivered to the tumor, limiting efficacy of the ADC and increasing the likelihood of off-target toxicity. This instability can be avoided by hydrolysis of the maleimide after the formation of the thiosuccinimide, which results in ring opening and an irreversible linkage. This can be accomplished through high pH and high-temperature incubation of the ADC after conjugation [37], or through the use of “self-hydrolyzing” maleimides [38]. Maleimide stability can also be modulated by the choice of attachment site, as maleimide stability is conjugation-site-dependent, with thiol pKa and solvent accessibility having a direct impact on propensity for deconjugation and hydrolysis [39,40].

Advancements in linker design are key contributors to the clinical success of ADCs. Linkers must be able to keep the cytotoxic payload stable in plasma, but then must also facilitate rapid and efficient release of the active drug in tumor cells. Both cleavable and noncleavable linkers have been successfully employed in approved ADCs (Table 1). Payloads that require release of the unmodified free drug upon internalization in the tumor cell to maintain drug potency require a cleavable linker. Cleavable linker technologies most widely used are reducible disulfides, acid labile-hydrazones, and protease cleavable dipeptides [10]. Upon internalization by receptor-mediated endocytosis, ADCs enter the endosomal to lysosomal pathway, where they are exposed to changing cellular conditions. The acidic environment of the late endosome promotes drug release from pH-sensitive hydrazone linkers, while disulfide linkages are cleaved by reduction due to the high concentration of glutathione present in tumor cells [10]. One of the earliest linkers, the acid-labile hydrazone linker in gentuzumab ozagamicin (Mylotarg^®^), was plagued by instability in circulation and premature drug release, resulting in higher rate of fatal toxicity in a Phase 3 trial, and a voluntary market withdrawal in 2010 [13]. This early failure highlighted the need to use and develop more stable linkers with release mechanisms more specific for the endosomal/lysosomal pathway.

Peptide linkers rely on lysosomal proteases, such as cathepsins, for linker catabolism and payload release, and are more serum-stable than their hydrazone and disulfide counterparts. The most successful of these protease cleavable linkers is the valine-citrulline (val-cit) linker used in brentuximab vedotin, enfortumab vedotin, polatuzumab vedotin, and most recently in disitamab vedotin, which was granted Breakthrough Therapy designation by FDA in September of 2021 (Table 1). Developed by Seagen, this linker incorporates a *para*-aminobenzyloxycarbonyl (PABC) spacer after the peptide linker that is capable of self-immolation following cleavage of the dipeptide, resulting in release of the free, unmodified payload [10]. It has been used most frequently with the microtubule inhibitor monomethyl auristatin E (MMAE), which, when released as a free drug, maintains its nonpolar properties, allowing it to diffuse across membranes and exhibit a bystander effect on neighboring cells.

ADCs that use a noncleavable linker rely on complete degradation of the antibody in the late lysosomal compartment for payload release. One such example is the SMCC linker used in trastuzumab emtansine. This heterobifunctional linker utilizes N-hydroxysuccinimide (NHS) for attachment to lysine side chains of the antibody and a maleimide for attachment to the payload, L-DM1, which contains a free sulfhydryl. After proteolytic degradation of the antibody in the lysosome, the payload is released as lysine-MCC-DM1, which maintains its microtubule inhibition activity despite the free DM1 payload not being released [41]. The polarity of an amino-acid-derivatized linker-drug resulting from a noncleavable linker cannot exhibit a bystander effect, and its activity is reserved for cells expressing the target antigen that are accessible by the ADC. In this way, ADCs with noncleavable linkers may have less efficacy on solid tumors where tumor penetration is a challenge or where target antigen expression is heterogeneous. Despite these drawbacks, ADCs with noncleavable linkers enjoy a high degree of serum stability, and thus may have better safety profiles and reduced toxicity [41]. In all of these release mechanisms, the ADC is dependent on the cell to facilitate release of the payload, a unique attribute of this platform that adds to the complexity of these therapeutics.

#### 2.1.4. Future Direction

With almost 300 ADCs in preclinical/clinical development, the full potential of this technology is only beginning to be realized. Decades of research and clinical experience have improved our mechanistic understanding of these complex molecules, further defining the necessary characteristics of each of the modular components and enhancing our understanding of how tuning any one of these will affect the efficacy and tolerability of the final ADC. These learnings have enabled extension of this platform beyond oncology, with sights set on indications such as targeting autoimmune disorders through selective delivery of anti-inflammatories and microbial infections through delivery of potent antibiotics [42]. The emergence of new targets, novel payloads, advanced site-specific conjugation technologies, alternative antibody formats, and improved linkers in research and clinical development will no doubt enable a new generation of these targeted therapeutics, poised to increase the therapeutic window over existing drugs. The field has collectively demonstrated an ability to learn, innovate, and adjust based on clinical and research findings at a stunning pace, with patients ultimately reaping the benefits of this highly competitive area of development.

### 2.2. Antibody–Oligonucleotide Conjugates

Antibody–oligonucleotide conjugates (AOCs) are gaining momentum as a class of therapeutics with great potential, in no small part due to their ability to leverage advances in the field of ADCs over the last 10–15 years. Similar to ADCs, the three main components of an AOC are the antibody, the oligonucleotide payload, and the chemical linker connecting the two, and, similar to ADCs, there are of course numerous considerations for each of these components. However, unlike ADCs, the field of AOCs is relatively new and the impact of each of the components on overall efficacy and TI are less well understood than for ADCs. Because AOCs share similarities with ADCs with regard to their structure, methods of production, and downstream analytics, AOCs are viewed as highly developable, with the hopes for rapid advancement into the clinic. Just as importantly, AOCs are showing promise for targeting cell types and diseases where ADCs and oligonucleotides alone have not yet been successful [43].

#### 2.2.1. Payload

**Figure 2:**
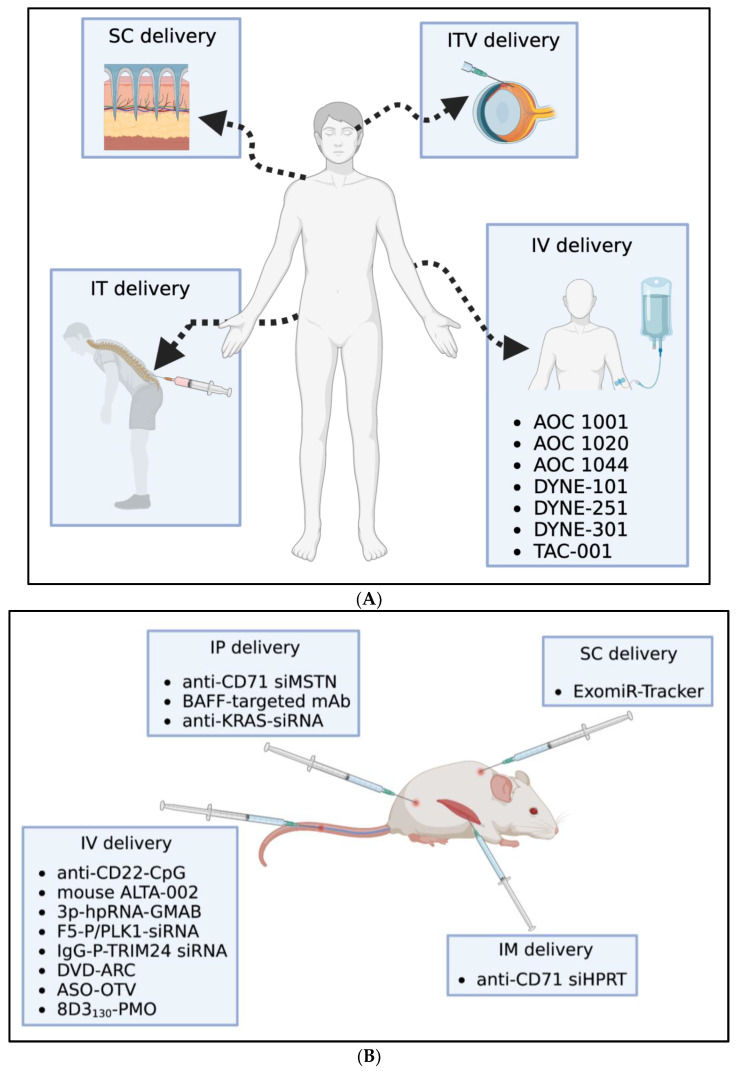
Routes of administration that have been used for (**A**) oligonucleotides and AOC delivery in the clinic and (**B**) for preclinical AOC delivery (IP: intraperitoneal, IM: intramuscular, IV: intravenous, IT: intrathecal, ITV: intravitreal, SC: subcutaneous). Created with BioRender.com.

Oligonucleotide therapeutics represent a very promising strategy for targets previously thought to be unattainable. By mimicking short RNA, oligonucleotides are able to act at the genetic level, thus blocking production of unwanted proteins before it begins. Moreover, oligonucleotides can also be designed to selectively target altered splicing patterns, which has opened up the possibility for therapeutics with exquisite selectivity [44]. Although oligonucleotides have been around for many years already and despite promising clinical developments with antisense oligonucleotide (ASO) [44,45], phosphorodiamidate morpholino oligonucleotide (PMO) [46], and small interfering RNA (siRNA) molecules [47], concerns have remained regarding poor PK and biodistribution profiles. Nonetheless, recent medicinal chemistry advances, along with expansion of oligonucleotide formats, have renewed the excitement in their potential once again [48,49,50]. Because an intravenously dosed oligonucleotide must overcome numerous biological barriers in order to reach its intracellular target, it is not surprising that many of the clinically advanced or approved oligonucleotides are either leveraging alternative administration routes such as subcutaneous (SC), intrathecal (IT), and intravitreal (ITV) injection [50] or are being paired with a delivery strategy for intravenous delivery (IV) (Figure 2A) [51,52,53]. Thus, more recently, the focus of oligonucleotide development has expanded to include therapeutic delivery, in addition to oligonucleotide design considerations.

As mentioned above, oligonucleotides face many hurdles upon administration. The first challenge is susceptibility to degradation in circulation. While chemical modifications have dramatically improved the stability of oligonucleotides, there is nonetheless a wide gamut of behavior, depending on the oligonucleotide design. For example, there are already a multitude of approved ASOs as well as ones in the clinic being administered through a variety of routes of administration as standalone therapeutics (Figure 2A) [53]. Similarly, PMOs have proven to be successful for targeting specific RNA splice sites, with several approved without the need for a delivery component [46]. On the other end of the spectrum, it is well known that naked siRNA cannot penetrate cell membranes alone and therefore requires a delivery strategy for therapeutic efficacy [52].

The next challenge faced by oligonucleotide therapeutics is that of distribution to the desired target, which can be broken into two separate components: tissue-specificity and endosomal escape. Although systemically administered ASO and siRNA tend to be most highly accumulated in tissues that are rich in reticuloendothelial cells, including the liver and spleen, or in kidney proximal tubule cells, oligonucleotides have the capacity to enter most tissues (other than the CNS) to some extent [54]. To enhance extrahepatic delivery, many of the next-generation oligonucleotide therapeutics are being paired with various antibody-based formats for cell-specific delivery (Table 2, Figure 2B).

The second impediment to distribution is the ability of the oligonucleotide to be released to the desired intracellular compartment. Upon reaching the cell surface, internalization into the cell happens through endocytosis, followed by trafficking through multiple intracellular compartments. It has been well demonstrated that much of the oligonucleotide that accumulates in cells becomes nonproductively trapped in endomembrane compartments, and only a small amount of oligonucleotide can leach out to the cytosol and diffuse to its final destination [55,56]. It is this minor component of the total cellular pool that is pharmacologically active. Notably, even though phosphorothioate-based ASOs are taken up more effectively than either siRNA or PMO, they are still subject to endosome trapping.

A thorough review of the current literature revealed the types of oligonucleotides that have thus far leveraged conjugation to antibody-based formats, including siRNA, ASO, PMO, cytosine-phosphate-guanine dinucleotide (CpG), anti-microRNA (anti-miRNA), and 5′ triphosphate hairpin RNA (3p-hpRNA) (Table 2). These formats rely on three main mechanisms of action for therapeutic efficacy: (1) RNA inhibition (RNAi), which mimics a natural cellular process that silences gene expression (ASO, siRNA, anti-miRNA), (2) splicing modulation (PMO), and (3) immunostimulation (CpG, 3p-hpRNA). While all of these oligonucleotides are negatively charged, PMOs represent an exception to this rule, since they are uncharged molecules. As a standalone therapeutic, this may be an advantage, potentially reducing their nonspecific interactions with circulating proteins and improving their intracellular uptake by eliminating charge repulsion with anionic cell membranes. Finally, it is worth noting that although there are a couple of examples of approved aptamers [45,50], there have yet to be examples of aptamer–protein conjugates for intracellular delivery, and, thus, aptamers are excluded from this discussion.

#### 2.2.2. Linker

Similar to how the chemical nature of the payload, linker composition, and DAR are all factors that impact PK and clearance of ADCs, these must also be considered for their impact on the physicochemical properties of AOCs. However, unlike the field of ADCs that can leverage years of research into the influence of each component, the importance of a specific linker to the overall mechanism of action, efficacy, or TI is less well understood for AOCs. It is worth mentioning, nonetheless, that, although a feasible approach, noncovalent linkages are useful for analytical applications and screening, but are not suitable for therapeutic applications.

Currently, those programs that have already reached the clinic are using a variety of covalent conjugation strategies. In the case of Avidity, their AOC programs have opted for a noncleavable linker to ensure maximal delivery to cells [57]. On the other hand, as part of their FORCE™ platform, Dyne uses a well-known cleavable ADC linker val-cit, in order to achieve maximal modification of the target [57,58]. This linker is stable in plasma but will be enzymatically cleaved in the endosomal compartment to enable release of the oligonucleotide payload in the cytosol. In the case of Tallac, which also has an AOC in the clinic [59] and one that will enter soon [60], candidate development has involved incorporation of antibodies aimed at different targets, with various Fc engagement levels, linkers, and CpG payloads.

Within preclinical research, the types of linkers that have thus far been explored include maleimide-based linkages [61,62,63,64], multifunctional peptide linker [65], β-lactam [66], and NHS/ester/azide linkers [67]. Interestingly, most of the linkers used are not derived from ADC designs. In fact, in the study described by Sugo et al., they examined several types of linkers for covalent conjugation of an antitransferrin (anti-TfR1) antibody fragment (Fab) to siRNA and found that a maleimide linker (noncleavable) was effective, whereas cleavable linkers (such as val-cit and dimethyl SS linkers) did not improve silencing activity. Their data also suggested that low-molecular-weight antibodies and fragments have considerable advantages over full-length mAbs when applied to endosomal release. Interestingly, in Shi et al., the choice was made to explore a linkerless approach, where the 3p-hpRNA oligonucleotides were noncovalently complexed with a modified lupus autoantibody (GMAB). In that study, the same antibody also served to bind the ENT2 receptor, enabling cell membrane crossing [57].

**Table 2:** Summary of therapeutic oligonucleotide–antibody conjugates over the past 10 years, organized according to tissue target or indication.

Name	Oligo Type	Indication	Ab Properties	Tissue Target	Current Phase	Sponsor	Reference
AOC 1001	siRNA	DM1*	TfR1-targeting mAb	Muscle	Phase 1/2	Avidity	[68,69]
DYNE-101	ASO		TfR1-targeting Fab		Phase 1	DYNE	[70]
AOC 1044	PMO	DMD	TfR1-targeting mAb		Phase 1/2 announced	Avidity	[71]
DYNE-251	PMO		TfR1-targeting Fab		Phase 1	DYNE	[72]
AOC 1020	siRNA	FSHD	TfR1-targeting mAb		Phase 1/2 announced	Avidity	[73]
DYNE-301	ASO		TfR1-targeting Fab		IND	DYNE	[58]
anti-CD71 siMST	siRNA	PAD	TfR1-targeting mAb		preclinical	Takeda	[62]
anti-CD71 siHPR		N/A					
N/A	siRNA	MG	BAFF-targeted mAb		preclinical	University of Texas	[74]
TAC-001	CpG	Cancer	CD22-targeting mAb	B cells	Phase 1	Tallac	[59]
ALTA-002	CpG		SIRPα-targeting mAb	Dendritic cells	IND	Tallac/ALX Oncology	[60]
N/A	3p-hpRNA		Cell-penetrating mAb	Tumor cells	preclinical	Gennao Bio	[75]
ExomiR-Tracker	anti-miR		Exosome-targeting mAb	Tumor cells	preclinical	Nagasaki University	[76]
KRAS-siRNA–anti-EGFR	siRNA		EGFR-targeting mAb (cetuximab)	Tumor cells	preclinical	University of Muenster	[63,64]
F5-P/ PLK1-siRNA	siRNA		Her2 targeting ScFv-protamine fusion	Her2+ tumor cells	preclinical	Sun Yat-sen University/University of Science and Technology of China	[77]
IgG-P-TRIM24 siRNA	siRNA		PSMA-targeting mAb	Prostate tumor	preclinical	Fourth Military Medical University/Xi’an Jiaotong University	[78]
N/A	siRNA		EGFR-targeting mAb (cetuximab)	Tumor cells	preclinical	Tianjin Medical University	[65]
DVD-ARC	siRNA		BCMA-targeting DVD	MMC	preclinical	Scripps/Alnylam	[66]
ASO-OTV	ASO	N/A	TfR1-targeting mAb	Brain	preclinical	Denali/Secarna	[79]
N/A	PMO	SMA	TfR1-targeting mAb		preclinical	University of Oxford/AstraZeneca	[61]
N/A	dsASO	Glioblastoma	CD44-, EphA2-, EGFR-targeting mAbs		in vitro	University of Toronto	[67]

BCMA: B-cell maturation antigen; CMV: cytomegalovirus; DM1*: myotonic dystrophy type 1; DMD: Duchenne muscular dystrophy; dsASO: double-stranded ASO; EGFR: epidermal growth factor receptor; FSHD: facioscapulohumeral muscular dystrophy; MG: myasthenia gravis; miR: microRNA; MMC: multiple myeloma cells; N/A: not available; OTV: oligonucleotide transport vehicle; PAD: peripheral artery disease; SMA: spinal muscle atrophy.

#### 2.2.3. Antibody

Similar to for ADCs, addition of an mAb can enhance the efficacy and TI of oligonucleotide-based medicines by stabilizing the oligonucleotide in circulation against nuclease activity, by extending half-life through neonatal Fc receptor recycling (FcRn), by conferring selectivity to a cell-surface receptor associated with the target of interest, thereby increasing cell penetration or barrier transcytosis, and by decreasing off-target effects and increasing bioavailability. While AOCs hold great promise, none have yet been approved; thus, they are not yet a well-validated therapeutic strategy. Nonetheless, two companies have already emerged as leaders in the field, Avidity and Dyne, both with AOC candidates in the clinic. Both companies have likely been able to advance rapidly owing to their ability to leverage already existing conjugation platforms. Interestingly, both of these companies, along with a few others, are aligned in their scope: delivery to muscle tissues using transferrin receptor 1 (TfR1) targeting for cell entry.

Thus far, anti-TfR1 antibodies appear to be the most popular choice in AOC design (Table 2). TfR1, also known as cluster of differentiation 71 (CD71), is a type II transmembrane glycoprotein that binds transferrin (Tf) and performs a critical role in cellular iron uptake through the interaction with iron-bound Tf. Since iron is required for multiple cellular processes and is essential for DNA synthesis and cellular proliferation, TfR is present on nearly every cell type, making it a relatively obvious choice as an antibody target. Additionally, since iron is highly trafficked to muscle cells, targeting this receptor is an ideal strategy for delivery to muscle tissues [62]. Moreover, Tf and TfR play an important role during the uptake and transcytosis of iron through blood–brain barrier (BBB) endothelial cells (ECs), and thus it is not surprising that the Tf pathway is also being considered as a key target for BBB transcytosis and delivery to the CNS [61,80,81]. Although there are only preclinical data available, the hypothesis has been proposed that tissue-specific TfR1 targeting can be achieved by varying the antibody’s affinity for TfR [61,82].

Avidity’s AOC conjugation platform leverages full-length, effector-null mAbs designed to target muscles for delivery of siRNA into the cytoplasm and nucleus. Recently, they announced positive Phase 1/2 data, reporting meaningful target reduction in 100% of participants using AOC 1001 [69]. On the other hand, Dyne’s FORCE™ platform is using a Fab format instead of a full-length mAb, to avoid FcRn-mediated recycling and because Fab binding does not impact receptor surface expression, internalization, or degradation [83]. Since Avidity and Dyne appear to be at a comparable stage in their clinical development, with very few obvious differences, differentiation in the clinic will likely be through more subtle differences in design of the oligonucleotide payload, linker chemistry, and target affinity.

Other, less traditional, antibody formats are also being considered for oligonucleotide delivery. For example, in vivo preclinical studies with compelling research include the Scripps/Alnylam collaboration using a dual-variable domain antibody (DVD) for increased half-life. In their study, they compared an siRNA that was chemically stabilized with a trivalent N-acetylgalactosamine (GalNAc) ligand to a DVD–siRNA conjugate. After IV injection in mice, they measured a half-life of 1.9 h for the GalNAc-siRNA versus 10–12 h for the DVD-ARC, the latter being the mean from two independent studies. Furthermore, Denali has also shown good CNS penetration with their BBB TV (transport vehicle): an engineered Fc domain that binds to TfR1 and has enhanced brain uptake and pharmacodynamic response of protein therapeutics in mouse and nonhuman primates [84,85]. Although many of the molecules that are currently in their pipeline, in clinical development and earlier, are taking advantage of a fusion protein format, their collaboration with Secarna appears to be aimed at developing AOCs.

### 2.3. Peptide–Oligonucleotide Conjugates

Peptide–oligonucleotide conjugates are an intriguing alternative to AOCs. While some of the same features as antibody conjugates are present, such as the ability to aid with circulating stability [86], and cell entry [87], there are also some distinct advantages. For example, pairing an oligonucleotide with a positively charged, cell-penetrating peptide (CPP) enables a dual role for the peptide: (1) to complex the negatively charged oligonucleotide and, as its name suggests, (2) to penetrate cell membranes for intracellular delivery (Table 3).

Taking advantage of the charge–charge interaction enables delivery of the oligonucleotide without the need for a linker, thereby decreasing the complexity of the system and increasing the potential for free API to be released intracellularly. To date, protamine and tat CPPs have been most commonly employed for these purposes (Table 3) [100,101]. Moreover, in some cases, the CPP has been fused to an antibody-based format to impart additional targeting capabilities. Such is the case in Song et al. [96] and Peer et al. [99], where the antibody enabled cell specificity without covalent conjugation of the oligonucleotide. Finally, it can be surmised that the relatively small molecular size and ability to be synthesized synthetically position peptide–oligonucleotide conjugates to capitalize on relatively straightforward chemistry, manufacturing, and controls (CMC). Nonetheless, this class of therapeutics is not without its challenges. Although publications date back to the beginning of 2000, the field does appear to have progressed beyond preclinical studies; thus, there appears to be a long road ahead. This is likely due to the plethora of unknowns and safety risks associated with CPPs, as well as not being able to leverage ADC learnings from the clinic, as is the case with AOCs.

Overall, this is a very exciting time for the field oligonucleotide–protein/peptide conjugate therapeutics. The more recent medicinal chemistry advances have led to an explosion of oligonucleotide formats, and the ability to combine these with well-validated linker chemistry and antibodies has led to very fast development timelines. Many factors must be taken into consideration, such as the mechanism of action and reactivity of the oligonucleotide, the structure of the antibody, and affinity for its target in order to tailor the conjugate to the therapeutic application or specific tissue/cell type. One area that will be interesting to watch evolve is that of systemic delivery to the CNS. While there is a lot of potential for oligonucleotide-based therapies to treat glioblastomas and neurodegenerative diseases, crossing the BBB presents yet another biological barrier that they must overcome. With Denali and Secarna announcing the expansion of their partnership over a year ago, the next few years are sure to bring exciting developments, and the findings from these and other studies will pave the way for a better understanding of the most important AOC design considerations.

### 2.4. Protein Conjugate Vaccines

Vaccines exploit the ability of the evolved adaptive immune system to respond to, and remember past encounters with, pathogen antigens [102]. A vaccine is used to safely induce an immune response that confers protection against infection and/or disease from subsequent exposure to a given pathogen. An effective vaccine must contain antigens that are either derived from the natural pathogen itself or produced synthetically to represent the biological components of the pathogen to the immune system. Most vaccines are composed of one or more protein antigens that induce immune responses that provide protection. However, polysaccharide antigens can also induce protective immune responses and are the basis of vaccines that have been developed to prevent several bacterial infections, such as pneumonia and meningitis caused by *Streptococcus pneumoniae*, since the late 1980s. Protection conferred by a vaccine is measured in clinical trials that relate immune responses to the vaccine antigen to clinical end points (such as prevention of infection, a reduction in disease severity, or a decreased rate of hospitalization). For some pathogens (e.g., *Haemophilus influenzae* serotype b [Hib], a long-time common cause of bacterial meningitis in young children), the immunogenic agent of the pathogen is a polysaccharide rather than a protein [103,104,105]. This has traditionally presented a hurdle for vaccine development, as polysaccharides are T-cell-independent antigens and lead to primarily IgM responses with little to no class-switching to IgG. As such, vaccination with polysaccharides fails to elicit robust immunogenic memory and booster response in the context of repeated exposure as compared to vaccination with protein antigens [106,107]. This effect is even more pronounced in young children (<18 months), where direct immunization with the HiB antigenic carbohydrate polyribosyl ribitol phosphate (PRP) [108] failed to produce protective levels of anti-PRP antibodies in a clinical trial of 100,000 individuals aged 3 months to 5 years [106,109]. In the late 1980s, conjugate vaccines were developed in which the PRP was covalently linked to a protein carrier. Similar to the strategy of traditional adjuvants, these protein conjugate vaccines are able to elicit T-cell-dependent responses in infants aged six to eight weeks [110] and the protein component encourages class switching from IgM to IgG via T-helper cells [111], conferring long-term immunity. The success rate for this strategy was very high in the case of Hib (>95%), and similar success in immunization with conjugate vaccines had been found for other bacterial pathogens such as *Salmonella typhi, Neisseria meningiditis,* and *Streptococcus pneumoniae* [104,105,112]. In the case of *Salmonella enterica*, which causes the potentially fatal typhoid fever, two traditionally available vaccines consisting of either a live attenuated form of the bacterium (Ty21a) or the purified capsular polysaccharide Vi (ViCPS) have been used extensively, although these are limited by being unsuitable or not immunogenic enough for children, as was seen in the case of Hib. Recently, a newer typhoid conjugate vaccine comprising Vi conjugated to tetanus toxoid (Typbar TCV) demonstrated promising immunogenicity and safety results in the clinic, earning the vaccine World Health Organization (WHO) prequalification in 2018 [112].

#### 2.4.1. Selection of Protein Carriers

Various protein carriers have been utilized in protein conjugate vaccines over the years. These include tetanus toxoid (TT), *Haemophilus influenzae* protein D (PD), outer membrane protein complex of serogroup B meningococcus (OMPC), diphtheria toxoid (DT), and CRM_197_ (an attenuated form of *C. diphtheriae* toxin featuring a single glycine to glutamic acid mutation that greatly reduces toxicity) [113,114]. In particular, diphtheria and tetanus toxoid were selected for use in Hib conjugate vaccines as they both have an ample body of established clinical evidence supporting their safety as vaccine antigens [113]. PD is derived from nontypeable *Haemophilus influenzae* (NTHi) and has been used as the carrier for a variety of serotypes in multivalent pneumococcal conjugate vaccines [115], including Synflorix and PHiD-CV (see below). OMPC has been similarly used in Hib and pneumococcal conjugate vaccines [116,117]. In the case of *Streptococcus pneumoniae*, the first pneumococcal polysaccharide-based vaccine (PPV) was licensed in 1983 and covered 23 serotypes (PPV23, Pneumovax 23). Owing to the above limitations of poor immunogenicity in children and inability to induce immune memory, the first pneumococcal conjugate vaccine (PCV) was designed to cover seven of the most common serotypes (PCV7, Prevnar) and was licensed in the United States in the year 2000. Subsequent vaccines expanded coverage to 10 or 13 serotypes (PCV10, Synflorix and PCV13, Prevnar 13, respectively) [118,119]. For such polysaccharide–protein conjugate vaccines, the polysaccharide components can be harvested individually from serotypes grown in culture medium, purified by standard physical and chemical methods, and then chemically coupled to the chosen carrier protein. In the case of marketed PCV7 and 13 vaccines, the bacterial polysaccharides are chemically activated and directly conjugated to the attenuated diphtheria toxin protein CRM_197_ by reductive amination to yield the glycoconjugate [118,120]. Subsequent development and clinical trials have led to additional pneumococcal PCVs with improved serotype coverage appearing on the market (Table 4).

One potentially beneficial consequence of conventional carrier protein choices—for example, TT, DT, PD, etc.—is that these conjugate carriers will elicit an immune response against their cognate pathogens in addition to that of the polysaccharide target. This can be leveraged in cases where additional immunity to the carrier protein may be of additional benefit to the therapeutic outcomes of treatment. For example, NTi PD was chosen as the carrier protein in Synflorix, and as the carrier for eight of the ten polysaccharides in the pneumococcal conjugate vaccine PHiD-CV, out of a desire to also confer immunity to NTHi, which is commonly associated with infection of the middle ear following pneumococcal respiratory infection, particularly in infants [121]. In other cases, the carrier molecule and polysaccharide component may be derived from the same pathogen as a means to trigger enhanced immunity. This has been demonstrated in vaccine conjugates of pneumococcal pneumolysin protein with polysaccharides from the same [122], as well as in vaccines derived from recombinant *Staph. Aureus* protein Hla conjugated to either *S. aureus* poly-*N*-acetylglucosamine (PNAG) or type 5 capsular polysaccharide. These conferred immunity to both the protein and saccharide components of these pathogens in mice and rabbits, respectively [123,124].

Additionally, novel candidates for conjugate vaccine carrier proteins have been explored in recent years. Recombinant forms of the *Pseudomonas aeruginosa* protein exotoxin A (*r*EPA) have been used as carriers for *S. aureus* type 5/8 capsular polysaccharides [125] and *Salmonella typhi* Vi antigen [126,127] in both research and clinical settings. With a variety of carrier protein choices available for the development of conjugate vaccines, the choice of which molecule to use should be based on considerations such as purity, ease of production, physicochemical properties, stability, and safety profile, as well as capacity to elicit a robust and effective immune response when coupled with the target antigen. Additional consideration to novel carrier proteins may be warranted if their properties exhibit preferable characteristics or facilitate different strategies for a given vaccine target compared to more conventional choices.

#### 2.4.2. Next-Generation Approaches for Improved Conjugate Vaccines

##### Site-Specific Conjugation of Polysaccharides to Protein Carriers

In spite of clinical successes, there are certain drawbacks to conventional methods of producing protein conjugate vaccines. For example, in the case of chemical conjugation of polysaccharides to CRM_197_ and other carriers, the resulting conjugate can exhibit heterogeneity that may compromise the reproducibility of the drug product. Additionally, nontargeted chemical conjugates can carry the risk of masking T-cell epitopes, which in turn compromise the immunogenicity of the vaccine. Such an outcome may necessitate coadministration with an additional protein adjuvant, which can improve immunogenicity, but can also increase the risk of adverse safety events [128,129,130,131]. One approach to overcome this is to carry out the conjugation of the antigen to the carrier in a more targeted fashion. For example, CRM_197_ has been used for controlled functionalization with both a carbohydrate antigen and a small molecule immune potentiator via a process termed “disulfide rebridging”, whereby a specific disulfide bond in the carrier protein is selectively reduced and modified by reaction with a species that reforms the bridge while introducing an additional functional entity [132,133,134]. In the case of CRM_197_, double Michael addition of bromopyridazinedione derivatives has been used to introduce a variety of immunopotentiator compounds, including small molecule agonists of Toll-like receptors TLR2/6 or TLR7/8 [132,135,136,137]. While the development of such approaches is ongoing, further development of robust methods for controlled coupling of chemical adjuvants to carrier proteins has potential for great clinical impact [138,139].

Taking the idea of targeted conjugation further, some researchers have used non-native amino acids (nnAA) and click chemistry to generate a conjugate vaccine in which the pathogenic polysaccharide is directly conjugated to a conserved protein antigen from the same pathogen species [140]. Specifically, in developing a vaccine for the group A carbohydrate (GAC) and polyrhamnose core thereof (GAC^PR^) from *Streptococcus pyrogens* [141,142,143,144], investigators sought to use the autologous protein antigen streptolysin O (SLO) on the basis of it being a key virulence factor [142]. These studies used a *C*-terminal truncation of SLO previously known to elicit a neutralizing immune response in rodents [142] that was genetically modified to convert specific solvent-exposed lysine and/or arginine residues to the non-native amino acid residue p-azidomethyl phenylalanine (pAMF) and expressed by cell-free protein synthesis [140]. These modified carrier proteins were then reacted with a dibenzocyclooctyne (DBCO)-derivatized form of GAC^PR^ to yield glycoconjugate vaccines [140,145]. These conjugate vaccines produced by nnAA click chemistry were compared to conventional GAC^PR^-SLO conjugates produced by reductive amination, and it was found that only the former retained immunogenicity of the SLO carrier and, in turn, conferred antibody-mediated protection in vivo [140].

##### Noncovalent Conjugation for Modular Vaccine Generation

Certain challenges in a given protein conjugate vaccine system can arise from the sheer diversity of antigenic molecules needed to exhaustively offer immune protection to a given pathogen. For example, as noted above, in the case of *Streptococcus pneumoniae*, the commercial conjugate vaccines Prevnar, Synflorix, and Prevnar 13 target three different subgroups of the known *S. pneumoniae* serotypes. However, in reality, there are over 90 serotypes of *S. pneumoniae*, the full extent of which these commercial products cover only partially [146]. Recent work has sought to generate modular conjugation systems that may be used to conjugate a wide array of pathogen serotype carbohydrates onto a carrier molecule through the noncovalent, yet very high affinity, binding of the avidin–biotin interaction [146,147]. This work has focused on using recombinant variants of the non-serotype-specific pneumococcal surface proteins PsaA and PspA as the protein carriers, which are, in turn, recombinantly fused to modified streptavidin (SA) [146]. In this way, Psp/Psa-SA fusion proteins can be expressed and combined with biotinylated pneumococcal polysaccharides in any given combination to yield a conjugate vaccine containing any desired subset of the pneumococcal serotype antigens. Studies found that using biotinylated capsular polysaccharide of *S. pneumoniae* type IV (b-CPS4) noncovalently bound to Psp/Psa-SA was sufficient to produce a superior humoral and cellular immune response as compared to the protein antigen alone [146,147]. This strategy can be further extended to other antigenic carbohydrates, limited only by the availability or generation of biotinylated species [148,149] in the case of the biotin-SA system. This approach may be generally extendable to other carrier molecules and/or modular binding partners when suitable for development of a particular vaccine.

##### Bioconjugation

Another approach to improve the modularity of these vaccines is the production of so-called bioconjugates, in which glycoconjugates of carrier protein and antigenic carbohydrate are synthesized directly in a bacterial cell line [124,150,151,152]. Two of the most well-characterized glycosylation pathways that can be potentially harnessed for this application are the *N*-linked glycosylation system of *Campylobacter jejuni*, and the O-linked system of *Neisseria meningitidis* or *Shigela flexneri* [153,154,155]. In both of these systems, a bacterial polysaccharide is transferred from a donor molecule, undecaprenyl pyrophosphate (Un-dPP), onto a protein acceptor at a specific residue within a known glycosylation motif [153,156]. Both the *C. jejuni* and *N. meningitidis* glycosyltransferases can be functionally expressed in *E. coli*, allowing for modular utilization of these biological conjugation systems within a well-characterized cell type [157]. Recent work has strived to identify and optimize the minimal required sequences for targeting of the carbohydrate antigen to the recombinant protein carrier molecule [153]. Such metabolic engineering of bacteria strains to produce and export into the periplasm polysaccharides linked to carrier proteins has been established for some time, and some of these bioconjugate vaccines have been successfully tested in clinical trials [158].

##### Nanoconjugate Vaccines in Virus-like Particles (VLPs)

Virus-like nanoparticles (VLPs) are self-assembling nanoparticles with a similar structural organization to viruses. VLPs exhibit a mosaic and repetitive surface organization of protein subunits which aids in the activation of the immune response by promoting B-cell receptor aggregation and complement fixation [159,160]. VLPs can be used to display antigens either by genetic fusion in the case of protein antigens [161,162,163] or bioconjugation and/or chemical conjugation in the case of carbohydrate antigens [163,164,165]. The O-linked glycosylation system of *S. flexneri* was recently leveraged in concert with the split-protein conjugation system SpyTag/SpyCatcher, which makes use of the isopeptide bond formed spontaneously between specific lysine and asparagine residues in two split units of the *Streptococcus pyogenes* protein CnaB2. This allows for a carrier molecule recombinantly modified with SpyCatcher protein to form a highly stable amide bond with a carbohydrate chemically modified with the SpyTag peptide in as little as 1 h [166,167]. In work by Li and colleagues, fusion of bacterial surface O antigen polysaccharides (OPS) to the SpyCatcher sequence was conjugated by mixing in solution with bacteriophage AP205 or Q-beta VLPs fused to SpyTag to produce nanoconjugate vaccines [159]. The researchers demonstrated that these VLP particles were capable of inducing high-titer antibody responses and protection against subsequent infections in BALB/c mice [159]. Future work may seek to extend upon this idea by optimizing the construction and production of both the antigen and scaffold components of such modular systems to provide the best features for generation of nanoconjugate vaccines in the context of a specific target pathogen. Future clinical impact of VLP-based vaccine approaches can be expected, with at least one VLP vaccine against the mosquito-borne pathogen Chikungunya virus currently in Phase 3 clinical trials [168].

**Figure 3:**
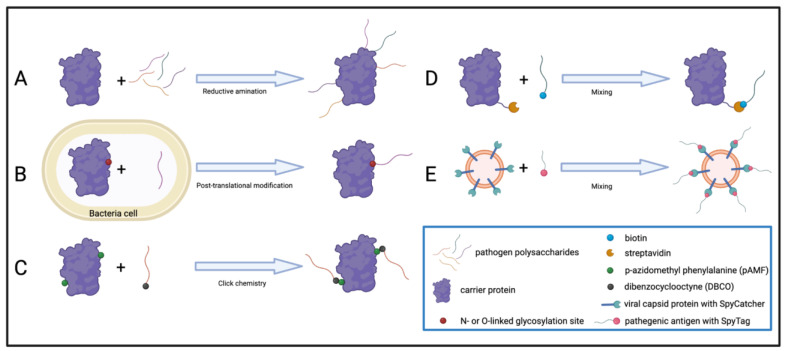
Schematic representation of protein conjugate vaccine approaches, demonstrating differences in geometry and valency in chemical conjugations, site-specific approaches, and VLPs, including (**A**) nonspecific chemical coupling, (**B**) bioconjugation, (**C**) click chemistry via non-native amino acids, (**D**) noncovalent modification using biotin and streptavidin, and (**E**) coupling to virus-like particles (VLPs). Created with BioRender.com.

Traditional vaccines preferentially use protein components of the target pathogen as antigens to elicit a strong humoral response from the adaptive immune system. In cases where protective immunity is dependent upon triggering an antibody response to non- or weakly-immunogenic components of the pathogen, such as carbohydrates, peptides, or small virulence factors, it may be necessary to combine these antigens with a protein carrier to achieve an antigen-specific immune response. While historical approaches have largely focused on leveraging traditional protein adjuvants to enhance inoculation with such antigens, an increasing number of modern-day approaches seek to utilize recombinant proteins and protein engineering to create a range of tools that can be applied to a broad range of challenges in the field (Figure 3). These novel approaches offer great promise for the future of engineered vaccines and their impact on public health.

## 3. Protein/Peptide Therapeutics That Are Enhanced through Chemical Modification

### 3.1. Introduction to Polymer–Protein Conjugates

Conventional polymer conjugates were developed to reduce immunogenicity and improve half-life of peptides and proteins with poor systemic PK. The increase in size afforded by polymer conjugation reduces renal filtration, while steric shielding by the polymer may allow proteins to resist opsonization, protease degradation, and antidrug antibody binding. The combined effect of these properties is a significant increase in the circulating half-life of polymer–protein and polymer–peptide conjugates, which sustains serum concentrations within the optimal therapeutic window for longer periods of time. The core principle in the design of polymer–protein conjugates is patient convenience; polymer conjugates are often designed as “biobetters”, leveraging established biology but decreasing the dosing frequency or improving the safety profile of existing therapeutics to enhance the overall patient experience during treatment.

An excellent case study exemplifying the benefits of polymer conjugation is pegfilgrastim, a PEGylated human granulocyte colony-stimulating factor (G-CSF) approved in 2002 for the prophylactic treatment of neutropenia during chemotherapy. Pegfilgrastim’s non-PEGylated predecessor, filgrastim, was limited by the short half-life of G-CSF, requiring a daily dosing regimen that placed a large burden on patients and healthcare systems. Covalent conjugation of a 20 kDa PEG to the *N*-terminus of filgrastim significantly extends its serum half-life (from a median half-life of 3.5–3.8 h with filgratism to 42 h with pegfilgratism) and enables administration of a single dose per chemotherapy cycle [169]. A retrospective comparison of pegfilgrastim and filgrastim use in breast cancer patients revealed that single-dose pegfilgrastim resulted not only in a lower patient burden, but improved therapeutic outcomes as well. A total of 72.4% of pegfilgrastim patients received their intended dose on time, compared to only 58% in the filgrastim group. Most notably, pegfilgrastim patients were nearly three times less likely to experience severe neutropenia [170]. The impact of pegfilgrastim on the healthcare system and patient burden is illustrated by its commercial success; pegfilgrastim recorded USD 69 billion in lifetime sales as of 2017, and six biosimilar products exist on the market today [171]. This narrative is mirrored in many other commercial polymer–protein conjugates; for example, weekly dosing of pegintron as a monotherapy in chronic hepatitis C patients led to a twofold higher incidence of a durable, complete response compared to the administration of the native IFN-α2b three times weekly [172]. Similarly, pegylation of asparaginase markedly reduced the frequency of hypersensitivity reactions in patients. As a consequence, although the cost per vial is higher for pegasparaginase, its superior safety profile leads to similar total payer costs relative to native asparaginase and a better treatment experience for patients [173].

Over the past few decades, monoclonal antibodies have comprised an increasingly large proportion of the biologics market. This has arguably reduced the need for systemic half-life extension through polymer conjugation, as FcRn recycling gives these molecules long circulation times with half-lives ranging from 6–32 days [174]. Genetic fusion of Fc, albumin, or albumin-binding domains have also become popular methods for extending the half-life of nonantibody proteins, such as cytokines and enzymes [175]. Nevertheless, the clinical landscape is shifting, with a greater emphasis on tissue-specific drug delivery, a need to access targets once considered “undruggable”, and a demand for new approaches to further reduce the treatment burden on patients and healthcare systems. Concurrently, advances in polymer–protein conjugates have revealed new functionalities beyond increased size that position them to have a broader impact on the design of next-generation, chemically enhanced peptides and biologics. In this section, we will discuss how advances in polymer chemistries, polymer architectures, and conjugation chemistries have revealed diverse applications for the design of novel polymer–protein and polymer–peptide conjugates.

### 3.2. Polymer Selection

#### 3.2.1. PEG

PEG has an extensive history in protein formulations, with >1000 approved therapeutics using PEG as an excipient. The first PEGylated protein, Adagen, was approved in 1990, giving PEG conjugates more than 30 years of clinical experience [176]. Adagen consists of a PEGylated bovine enzyme, adenosine deaminase (AD). Non-site-specific attachment of a 5 kDa PEG onto the enzyme both increased the half-life of AD and decreased immunogenicity against the nonhuman protein [177]. Many product approvals have followed, each of which employ increased half-life and/or reduced immunogenicity as central design principles (Table 5). In addition, PEGylation may improve the physical stability of proteins, including their solubility [178,179,180], colloidal stability [181], and melting temperature (T_m_) [182,183,184,185,186]. Today, PEGylated proteins still comprise the majority of clinical polymer conjugate candidates. Despite this widespread use, PEG is known to have several drawbacks that have motivated a wealth of research into novel polymeric alternatives to PEG. Among these drawbacks include its polydispersity, lack of biodegradability, and potential immunogenicity.

Mounting evidence suggests the possibility of immunological responses to PEG such as allergic reactions and formation of anti-PEG antibodies (APAs), which may impact the toxicology profile or efficacy of a therapeutic. A subset of patients experience hypersensitivity reactions such as anaphylaxis and infusion reactions after administration with PEG-containing formulations. Furthermore, up to 72% of the human population may have pre-existing anti-PEG antibodies, although reported prevalences vary widely depending on the assay used [187,188]. In most cases, the relatively low concentrations of APAs prevent them from impacting efficacy, but low-dose therapeutics may suffer from accelerated blood clearance (ABC) initiated by anti-PEG antibodies. In a Phase 2 trial of pegylated uricase (pegloticase), 41% of patients developed high-titer antibodies against the PEG portion of pegloticase, resulting in lower serum concentrations of pegloticase and a poor response to treatment in these patients [189]. Similarly, serum anti-PEG antibody levels were associated with a loss of PEG-asparaginase activity in acute lymphoblastic leukemia patients [190]. The increasing exposure to PEG in therapeutics may also lead to increasing overall APA levels in the human population over time; for instance, a small study of patients receiving the COVID vaccines revealed that the PEG-containing mRNA-1273 vaccine increased plasma anti-PEG IgM and IgG antibody concentrations 68.5- and 13.1-fold, respectively [191]. More research is required to fully understand the impact of repeated exposure to PEG-containing products on the safety and efficacy of these therapeutics.

**Figure 4:**
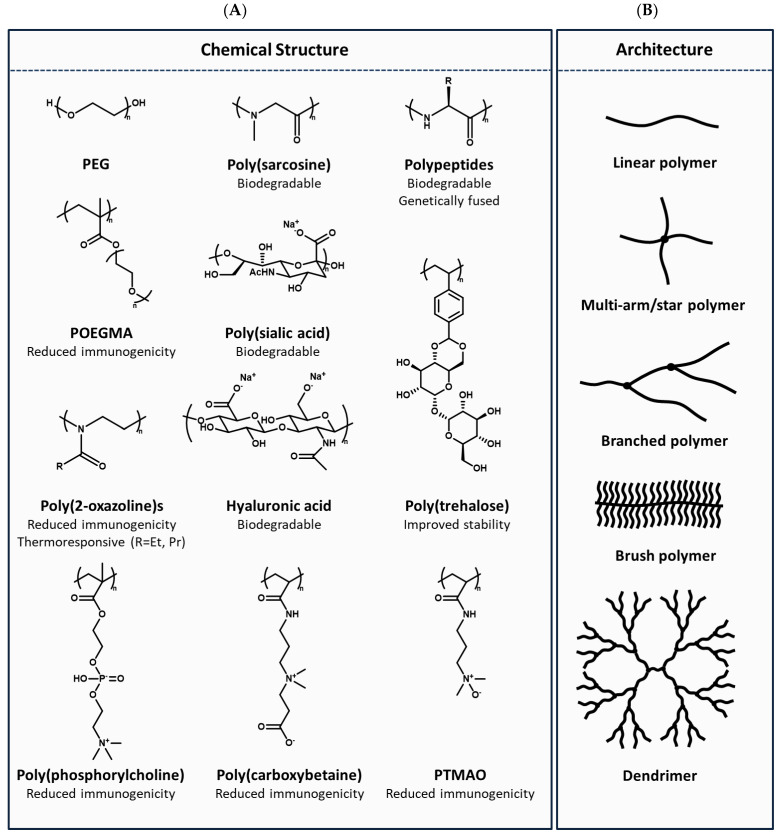
Overview of polymer (**A**) chemical structures and (**B**) architectures discussed in this section.

PEG is not biodegradable, resulting in an upper limit on the half-life extension achievable and potential concerns over its ability to accumulate in the body. PEGs up to 40 kDa have been used in the clinic, which is close to the renal filtration limit for PEG (roughly 50 kDa) [192]. PEGs in this size range also accumulate in the form of vacuoles in cells, but vacuolization has not been associated with any safety concerns in currently marketed therapeutics [193]. Finally, PEG is synthesized by ring-opening polymerization of ethylene oxide. The resulting product is polydisperse, which increases analytical complexity in both the PEG intermediate and the resulting conjugate. In addition, commercial PEGs are only functionalized at their termini, limiting their drug-loading capacity. These challenges have paved the way for the development of next-generation polymer chemistries (Figure 4A) and architectures (Figure 4B).

#### 3.2.2. Next-Generation PEG Derivatives

PEG derivatives with modified architectures have been proposed as potential alternatives to linear PEGs. For instance, the use of multiarm PEGs allows multiple APIs to be loaded onto a single polymer. Branched architectures may have more favorable PK properties as well. In one example, comb-shaped PEG polymers (“PolyPEG”) were prepared by grafting pendant PEG chains onto a polymethacrylate backbone. Despite their smaller hydrodynamic size, PolyPEG-IFNa conjugates had longer serum half-lives in rats; this effect was attributed to the modified architecture, which may allow the conjugate to further resist renal filtration or proteolytic degradation. In addition, PolyPEG conjugates were less viscous than the corresponding linear PEG conjugates [194].

Qi et al. extended this work to bottlebrush PEGs, a subclass of comb-shaped polymers characterized by very high graft densities [195] (Figure 4B). Dense bottlebrush architectures have been reported to be nonfouling, e.g., resistant to both cell and protein adsorption, which may enable them to evade APA recognition. Poly(oligo(ethylene glycol) methyl ether methacrylate) (POEGMA) bottlebrush polymer conjugates reduced APA-related immunogenicity and accelerated clearance; compared with two commercial PEG conjugates, Adagen and Krystexxa, POEGMA-exendin conjugates exhibited significantly lower binding to APAs in human plasma. Based on previous reports that the minimum antigenic PEG length is 6–7 ethylene glycol repeats, these authors further demonstrated that decreasing the average ethylene glycol side chain length to three eliminated APA reactivity entirely. These findings were then extended by Ozer et al. to include POEGMA conjugates with highly immunogenic uricase as a model protein. Administration of POEGMA–uricase conjugates eliminated both the accelerated blood clearance and the development of ADAs observed in groups treated with PEGylated uricase [196,197].

#### 3.2.3. Poly(2-oxazoline)s

Poly(2-oxazoline) (POZ) polymers represent promising alternatives to PEG, with reported benefits including low immunogenicity, low viscosity, high drug-loading capability, and oxidative stability. POZ polymers were shown to be significantly less viscous than PEG solutions at the same molecular weight with only a slight reduction in hydrodynamic size [198]. Lower drug product viscosity results in lower pain during injection for patients and enables the use of smaller diameter needles for administration [199]. POZ conjugates were successfully prepared with a variety of proteins, including BSA, insulin, and uricase, and the loss in bioactivity upon polymer conjugation was similar to the corresponding PEG conjugates. Additionally, repeat administration of POZ–BSA conjugates generated lower anti-BSA antibody titers in rabbits when compared with the PEG–BSA group, suggesting that POZ conjugates were more effective than PEG at shielding BSA immunogenicity [198].

POZ polymers can be synthesized with diverse properties; for example, POZs exhibit thermoresponsive behavior that is tunable by altering the monomer hydrophobicity. Poly(2-ethyl-2-oxazoline) has a cloud point temperature (T_cp_) of 61–69 °C, while the more hydrophobic poly(2-isopropyl-2-oxazoline) has a T_cp_ between 26–34 °C, and hydrophilic poly(2-methyl-2-oxazoline) is not thermoresponsive at any temperature [200]. In addition, conjugation handles can be incorporated directly into the polymer backbone during synthesis, allowing multiple drugs to be loaded onto a single polymer. Serina Therapeutics is in Phase 1 clinical trials with a POZ–rotigotine conjugate (SER-214), but to date, no large molecule POZ conjugates have entered the clinic.

#### 3.2.4. Zwitterionic Polymers

Zwitterionic polymers are gaining attention as alternatives to PEG for their ability to stabilize proteins against denaturation and evade immune recognition while maintaining extended circulation in vivo. These polymers contain an equal number of positive and negative charges to form net-neutral polymers that interact strongly with water, and their high degree of solvation is reported to impart ultra-low fouling properties [201]. Examples of zwitterionic polymers used to prepare polymer–protein conjugates include poly(carboxybetaine) (pCB), poly(phosphorylcholine) (pPC) [202], poly(sulfobetaine) (pSB), and poly(trimethylamine *N*-oxide) (pTMAO). KSI-301, a conjugate between an 800 kDa branched phosphorylcholine polymer and an anti-VEGF IgG, represents the most advanced zwitterionic polymer–protein conjugate in the clinic.

In the preclinical space, pCB conjugates have been prepared for a variety of therapeutically relevant peptides and proteins, including uricase, insulin, glucagon-like peptide-1, and IFN-α2a [203,204,205,206]. The pCB-IFN-α2a conjugates mitigated the accelerated blood clearance and partially restored the activity loss observed in the PEGylated counterpart, and pCB conjugation was also shown to protect ⍺-chymotrypsin from thermal and chemical denaturation. More recently, pTMAO conjugates have been reported as potential next-generation zwitterionic polymers. Similar to pCB, pTMAO conjugates have been shown to generate significantly lower anticonjugate antibody titers in vivo and eliminate accelerated blood clearance observed in the PEGylated controls. For example, pTMAO–uricase conjugates maintained extended serum half-life and sustained in vivo efficacy after three consecutive injections in mice. In contrast, PEG–uricase-treated groups exhibited lower serum half-lives and reduced efficacy after repeat injections, consistent with the loss of efficacy observed in the clinic for pegloticase. pTMAO conjugation was also shown to be superior to PEG in stabilizing uricase against thermal treatment at 50–70 °C and after incubation with urea.

Molecular dynamics simulations revealed that, relative to PEG, pTMAO accepted more hydrogen bonds from water per monomer, each hydrogen bond had a longer lifetime on average, and water formed a contiguous hydration shell around the polymer. All of these attributes likely contribute to the ultra-low fouling characteristics in this new class of polymer conjugates [207]. Nevertheless, additional work is required to understand the specific design features that enable zwitterionic polymers to be nonfouling. For example, the Leckband group recently found evidence that linear pSB polymers interact with certain proteins, leading to decreases in T_m_, while densely packed brush pSB polymers resist protein adsorption [208,209].

#### 3.2.5. Amino-Acid-Based Polymers

One of the most elegant approaches to reduce the CMC complexity of polymer–protein conjugates is the genetic fusion of unstructured polypeptides to the *N*- or *C*-terminus of the protein. A major advantage of this approach is that it allows for direct expression of the conjugate, substantially reducing the manufacturing complexity by eliminating several conjugation process steps. Notably, this strategy also enables the production of monodisperse, chemically defined conjugates, which simplifies analytical characterization. Finally, polypeptide fusion proteins are fully biodegradable, mitigating concerns about accumulation in vivo.

Examples of these polymers include XTEN (unstructured, hydrophilic, biodegradable protein polymers), proline/alanine-rich sequences (PAS), and elastin-like polypeptides (ELP). XTEN represents the most advanced format, with multiple programs currently undergoing clinical trials. XTEN polypeptides are constructed from hydrophilic amino acid building blocks (A, E, G, P, S, and T), and they are designed to have high chemical and physical stability, a lack of secondary structure, and high solubility and expression yields [210]. XTEN polypeptides exhibit properties of random coil polymers, allowing them to adopt larger hydrodynamic radii for a given molecular weight relative to globular protein. As a result, XTENylation achieves robust half-life extension for a series of peptides and proteins, with improvements in half-life ranging from 13–125-fold over their unmodified counterparts [211]. Similarly, PAS polypeptides are composed of P, A, and S amino acids and form hydrophilic, disordered polymers with no secondary structure. A PASylated anti-TNF-α Fab’ achieved similar half-life extension to the marketed PEGylated version, certolizumab pegol, while mitigating the antigen-binding loss observed in the PEG-conjugated antibody [212]. These disordered polypeptides can also be used as multivalent polymer scaffolds via the incorporation of reactive amino acids such as cysteine into the sequence, enabling the development of multivalent arrays with precisely defined ligand spacing [213] and high-DAR antibody–drug conjugates [214].

Inspired by tropoelastin, ELPs are polypeptide chains composed of VPGXG repeats, where “X” can be any amino acid except proline. ELPs are unique in their ability to undergo a temperature-dependent phase transition from a random coil structure to globular aggregates; when designed appropriately, this thermoresponsive behavior can be programmed to promote self-assembly and depot formation upon injection [215]. ELP fusions have been used to attain zero-order release of GLP1 over 14 days in nonhuman primates, a sevenfold increase relative to Trulicity, a marketed long-acting GLP1 therapy [216]. PB1046, an ELP fusion to vasoactive intestinal peptide, reached Phase 2 clinical trials before the program was voluntarily terminated due to COVID-19-related enrollment and manufacturing challenges [217].

#### 3.2.6. Dendrimers

Dendrimers represent a unique class of nearly monodisperse polymers, with compact structures, a high density of functional groups, and a large range of accessible surface chemistries. Dendrimers are primarily synthesized using the divergent approach, in which branched monomers are iteratively installed from a central core to form successive generations with exponentially increased branching (Figure 4B). The result is a dense and highly branched polymer with a high drug-loading capacity and physical properties that are largely governed by the identity of the terminal branches [218,219]. The use of a hydrophobic core enables noncovalent encapsulation of hydrophobic drugs, while surface functionalization enables targeting or multivalent display. Commonly used chemistries include poly(amidoamine) (PAMAM) [220], poly-2,2-bis(hydroxymethyl)propionic acid (bis-MPA) [221], and poly(L-lysine) dendrimers [222], but monodisperse, degradable PEG dendrimers have been recently reported as well [223,224]. Starpharma has several small molecule candidates in the clinic using their poly(L-lysine) dendrimer platform, but there are no protein or peptide dendrimer conjugates in the clinic to date.

#### 3.2.7. Biodegradable Polymers

A range of biodegradable polymers are being explored for the production of polymer–protein conjugates, including polysialic acid (PSA) [225,226], hyaluronic acid (HA) [227,228], and polysarcosine (pSar) [229]. Biodegradable polymers may address concerns over the potential accumulation of nondegradable polymers such as PEG in vivo, and they allow access to much larger size ranges while ensuring that these compounds can still be metabolized. HA and PSA are anionic biopolymers, while pSAR is a nonionic, hydrophilic polymer.

Polysialic acid is a biodegradable polymer of sialic acid, a component of cell membranes and glycoproteins. A PSA-rhFVIII recently completed Phase 1 clinical trials but was discontinued for lack of efficacy [230]. HA is an endogeneous polysaccharide that is degraded via hyaluronidases present in many tissues, including in the liver and kidneys. This semirigid polymer has a long estimated persistence length of 4 nm [231], giving it a significantly larger hydrodynamic size for a given molecular weight when compared with more flexible polymers such as PEG. HA is also internalized into cells through binding to CD44; this property has been exploited for delivery to CD44-overexpressing tumor cells [232,233].

Polysarcosine is a polypeptoid derived from sarcosine, an endogenous, noncoded amino acid. The polymer’s solution properties, including its solubility, hydrodynamic size, and interactions with proteins, are similar to those of PEG [234,235], but pSar-IFN-α conjugates exhibited lower antidrug antibody (ADA) levels and better antitumor efficacy than the PEGylated comparators after multiple administrations in mice [236]. Monodisperse, short pSAR chains have also been used to improve the physicochemical properties of ADCs [237]. This application will be discussed in more detail in a subsequent section.

#### 3.2.8. Trehalose-Based Glycopolymers

Proteins are susceptible to aggregation in aqueous formulations, and this physical instability often limits their shelf lives and storage temperatures. As many protein aggregates have been reported to be immunogenic [238], the levels of aggregates must be tightly controlled during the manufacturing and long-term storage of the drug product. The majority of therapeutic proteins must be stored frozen or refrigerated to maintain their physicochemical stability; these cold-chain requirements increase the cost and complexity of the supply chain and preclude global access to these therapeutics. Thus, strategies that permit the long-term storage of therapeutic proteins at room temperature remain highly desirable.

Inspired by commonly-used excipients such as trehalose and sucrose, the Maynard group demonstrated the ability of trehalose-based glycopolymers to protect proteins against environmental stresses such as thermal and agitation stress. Conjugation of the trehalose polymer stabilized lysozyme against successive lyophilization cycles, while conjugation to insulin stabilized the protein against agitation stress [239,240]. These properties may permit the removal of agitation-stabilizing surfactants such as polysorbates in poly(trehalose)-conjugated protein and peptide formulations, which are prone to instability and particle formation in aqueous formulations [241]. Similarly, lyophilized formulations may have greater flexibility to remove osmolality-increasing excipients such as monomeric trehalose or sucrose, which are included to protect against freezing and desiccation stresses during lyophilization. Multiple poly(trehalose) conjugates also showed improved stability during thermal stress at high temperatures, including 90 °C stress for lysozyme and insulin as well as 75 °C stress for Herceptin and its Fab fragment [242]. While these results are promising, no studies have evaluated the impact of polymer conjugation on protein stability at relevant storage temperatures, such as room temperature or 2–8 °C, where thermal unfolding and T_m_ are often poor predictors of stability [243]. This dataset will be needed to understand the broader potential of polymers such as poly(trehalose) to improve drug product shelf life.

### 3.3. Advances in Conjugation Chemistries

Advances in conjugation chemistries for the synthesis of polymer–protein conjugates are extensively covered in several excellent reviews [244,245,246,247] and will only be reviewed briefly here. Conjugation to peptides is relatively straightforward, as solid-phase peptide synthesis allows for the facile incorporation of functional handles into the peptide sequence. Thus, in this section, we focus the discussion on strategies for conjugating polymers to proteins.

#### 3.3.1. Grafting To

All commercial polymer–protein conjugates follow the “grafting to” approach to produce the final drug product (DP), in which the polymer is synthesized and functionalized prior to conjugation to the protein. This approach enables the use of mild, protein-compatible reaction conditions, but it often requires large molar excesses of polymer to drive conversion and the development of subsequent purification steps to remove the residual unreacted polymer. First-generation protein–polymer conjugates were prepared using nonspecific conjugation to lysine amino groups within the protein via PEGs terminated with activated esters such as NHS esters. This strategy typically generated heterogeneous conjugates with diminished activity due to the lack of control over the conjugation site [248].

Second-generation conjugates used more targeted, site-specific conjugation strategies to reduce heterogeneity and minimize activity loss. Rebinyn utilizes enzymatic conjugation with sialidase to conjugate a 40 kDa PEG to one of the two *N*-linked glycans in recombinant human factor IX [249]. Other conjugates were prepared through selective conjugation to the α-amino group on the *N*-terminus, which often has higher reactivity relative to lysine side chains due to its lower pKa. For example, pegfilgrastim is selectively PEGylated at the α-amino group on the *N*-terminus via reductive alkylation with PEG-aldehyde [250]. However, in many instances, including in mAbs, the *N*-terminus of the protein is in close proximity to the binding site. An alternative strategy to minimize the loss of binding in these molecules involves the introduction of engineered cysteines into the antibody sequence for conjugation to maleimides, pyridyl disulfides, or vinyl sulfones. Cimzia, a PEGylated Fab, incorporates an engineered cysteine at the *C*-terminus of the protein for site-specific conjugation to a maleimide-functionalized PEG via Michael addition [251].

Despite its prevalence in clinical and commercial conjugates, thiol–maleimide chemistry suffers from several CMC challenges, including gradual deconjugation and the potential for disulfide scrambling during the conjugation process. These shortcomings have motivated the development of next-generation, site-specific conjugation chemistries, which range from the use of noncanonical amino acids to enzymatic ligation onto specific recognition sequences engineered into the protein. Popular enzymatic conjugation methods include transglutaminase and sortase A. Transglutaminase catalyzes the formation of a stable isopeptide bond between a primary amine and a glutamine-containing sequence in the protein, while sortase A catalyzes the formation of an amide bond between a LPXTG sequence in the protein and an *N*-terminal oligoglycine [252,253,254,255]. While enzymatic approaches have shown promise for site-specific modification of proteins, the need to source an additional protein as an intermediate and subsequently purify it from the reaction mixture adds CMC complexity to the bioconjugation process.

The incorporation of noncanonical amino acids into the protein sequence significantly expands the repertoire of accessible conjugation chemistries. Since the pioneering work in this space by the Schultz lab, hundreds of non-natural amino acids have been incorporated into proteins during expression, enabling site-specific conjugation with a variety of biorthogonal chemistries [256,257]. In particular, noncanonical amino acids facilitate the use of click chemistries such as strain-promoted click chemistry; these bioconjugation reactions are advantageous as they typically proceed with rapid kinetics, high yield, and under mild conditions. SAR444245 is currently in Phase 2 clinical trials and uses a non-natural *N*^6^-(2-azidoethoxy)-carbonyl-L-lysine amino acid for site-selective conjugation to dibenzocyclooctyne-functionalized PEG. While the methods for incorporating non-natural amino acids into protein sequences are out of scope in this review, readers are referred to several recent reviews on this topic [258,259].

#### 3.3.2. Grafting From

While all commercial polymer–protein conjugates utilize the “grafting to” approach for conjugation, “grafting from” has recently emerged as an alternative to reduce purification process complexity and improve overall yields. This method uses protein-compatible controlled radical polymerization techniques, most commonly atom-transfer radical polymerization (ATRP) [260,261] or reversible addition-fragmentation chain transfer polymerization (RAFT) [262], to polymerize directly from the protein after the introduction of an initiator or chain transfer agent onto the protein. Because it uses small molecule initiators and monomers, the “grafting to” method can overcome steric barriers that may otherwise prevent the conjugation of a polymer to a protein, allowing for conjugation to sites with lower solvent exposure or denser packing of polymer chains near the protein surface. These monomers can also be readily separated from the protein via high-throughput purification techniques such as tangential flow filtration (TFF) [263].

Importantly, the “grafting from” strategy still requires modification of the protein with a reactive handle for polymerization; thus, the identification of site-selective conjugation chemistries remains vital to the successful development of polymer–protein conjugates. In addition, polymerization conditions must be carefully optimized to be compatible with proteins, which require the use of aqueous solvents and low temperatures; as a result, achieving a balance between mild polymerization conditions and low polymer dispersity is often challenging. Finally, ATRP uses transition metal catalysts such as copper, which is a potential concern as metal ions can bind to proteins and trigger chemical degradation, including oxidation and fragmentation [264,265].

### 3.4. Emerging Applications

Improved patient convenience through reduced dosing frequency remains a central goal in the design of protein– and peptide–polymer conjugates. Many clinical candidates continue to harness well-established design principles such as systemic half-life extension and improved immunogenicity as drivers for polymer conjugation (Table 6). This approach is most commonly employed to improve the properties of enzymes and cytokines, but emerging formats such as macrocyclic peptides are also limited by their short circulation half-lives and may benefit from polymer conjugation as well [266]. Thus, half-life extension through polymer conjugation is expected to continue to play an important role in the design of new peptide- and protein-based therapeutics.

**Figure 5:**
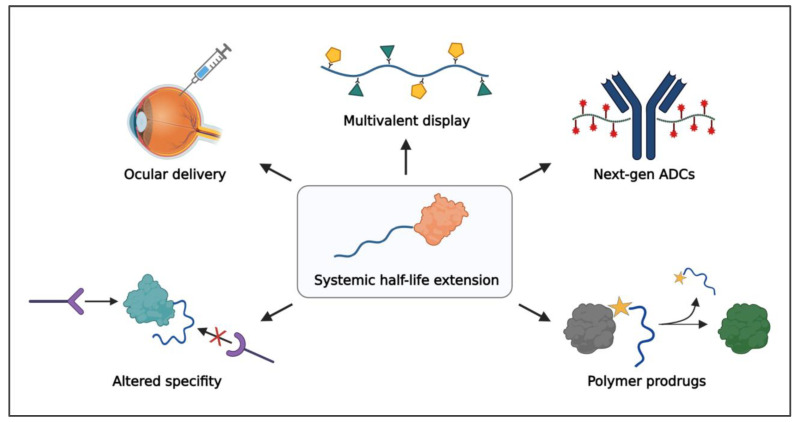
The diversifying applications of polymer–protein and polymer–peptide conjugates. While conventional conjugates were developed to improve the systemic half-life of peptides and proteins, recent work has demonstrated the ability of polymer conjugates to extend intravitreal half-life, alter the specificity of the conjugated protein, facilitate multivalent display of the API, improve the properties of ADCs, and enable the production of prodrug–polymer conjugates. Created with BioRender.com.

The development of novel polymers and conjugation chemistries has also paved the way for the next generation of polymer conjugates with diverse mechanisms of action beyond increased systemic half-life (Figure 5). Similarly, the expansion into tissue-specific delivery has revealed novel tissue targets for polymer conjugates that directly build upon concepts established for parenteral delivery. In this section, we discuss these next-generation applications of polymer–protein conjugates.

#### 3.4.1. Ocular Delivery

Sustained delivery of intravitreally-administered therapeutics is a rapidly growing field, and the delivery of polymer–protein conjugates is emerging as a key strategy for treatment of back-of-the-eye diseases such as neovascular AMD (nAMD), geographic atrophy (GA), and retinal vein occlusion (RVO). AMD is one of the leading causes of vision loss; in the United States alone, nearly 20 million adults are estimated to be living with AMD [267]. Decreased dosing frequency is critical in driving optimal therapeutic outcomes in these patient populations, as the need for frequent ITV injections at specialized clinics represents a significant patient burden. Fear of injections and an inability to secure transport to the hospital are among the cited reasons for nonpersistence rates as high as 60% after two years of treatment. Ultimately, poor adherence to the treatment regimen has led to inferior visual acuity outcomes for real-world patients compared with those treated in controlled clinical trial settings [268,269].

The development of polymer conjugates for sustained ITV delivery directly builds upon many of the design principles established for systemic half-life extension. The foundation of this approach is the recently established correlation between hydrodynamic radius and clearance from the vitreous humor [270], suggesting that macromolecule diffusivity is the primary driver of elimination from the vitreous. Importantly, binding to FcRn does not play a significant role in vitreous pharmacokinetics [271]. As a result, increased size through polymer conjugation has emerged as a key strategy to improve half-life in the vitreous for a variety of therapeutic proteins and small molecules.

Two intravitreally-delivered polymer conjugates are currently being evaluated in the clinic. Pegcetacoplan, consisting of two peptide C3 inhibitors covalently conjugated to each end of a 40 kDa linear PEG polymer, represents an extended half-life version of AL-78898A, which was terminated during Phase 2 GA trials. One of the two recent Phase 3 trials for pegcetacoplan met its primary endpoint for reduction in GA lesion size; if approved, pegcetacoplan will be the first polymer–protein conjugate marketed for intravitreal delivery and the first treatment for GA [32,272,273]. KSI-301, a conjugate between an anti-VEGF mAb and a branched phosphorylcholine polymer (pPC), is currently undergoing multiple Phase 3 trials for treatment of back-of-the-eye diseases [274]. While KSI-301 failed to meet the primary endpoint in the DAZZLE trial for treatment of nAMD, the company recently announced positive topline results in retinal vein occlusion (RVO). The increased hydrodynamic size afforded by conjugation to pPC resulted in noninferior efficacy in RVO while extending the dosing interval from monthly to every two months [275]. While the root cause for the failure of KSI-301 in the DAZZLE trial is unknown, one hypothesis is that the increased size prevented the conjugate from reaching the subretinal space, which may be important for efficacy in the nAMD patient population [276]. Thus, future programs evaluating polymer conjugates for the treatment of subretinal diseases may need to balance half-life extension through increased hydrodynamic size with adequate partitioning into the target tissue.

Other polymer conjugates have also been explored preclinically for half-life extension in the vitreous, including multiarm PEGs [277], PAMAM dendrimers [278], and HA conjugates [279]. For example, Famili et al. conjugated anti-VEGF Fabs onto HAs of variable lengths, generating multivalent arrays with hydrodynamic radii up to 29 nm, and achieved up to a 3.5-fold half-life increase in rabbit vitreous over the unconjugated Fab [280]. HA polymers possess many favorable properties for ocular delivery, including their large hydrodynamic size, lack of degradation in the vitreous, and rapid metabolism and clearance upon entering systemic circulation. Accordingly, Valitor has initiated preclinical development for an HA-anti-VEGF VHH conjugate for long-acting ocular delivery [281].

#### 3.4.2. Altered Binding Selectivity

The use of polymers to sterically block binding to specific epitopes on a protein is not new; polymer conjugates have attained widespread clinical success in preventing ADA binding and reducing immunogenicity for non-native proteins. However, a second emerging application expands this capability, utilizing polymers to alter the binding specificity of the conjugated protein itself. This approach has been primarily applied toward cytokines, which are a promising class of therapeutic agents but are often limited by their pleiotropy, leading to dose-limiting toxicity or undesirable secondary mechanisms of action. Selective polymer conjugation to cytokines has the potential to bias the activity of the protein without requiring modifications to its amino acid sequence.

Multiple clinical programs use PEGylation to alter the binding interactions between IL2 and its receptors. One class of IL2 therapeutics seeks to improve upon the properties of Proleukin, a marketed IL2 therapeutic for the treatment of metastatic melanoma and renal cell carcinoma. Proleukin treatment induces complete and partial responses in a subset of cancer patients, but its efficacy is limited by its narrow therapeutic window and its ability to expand immunosuppressive regulatory T-cell populations [282,283]. SAR444245 uses a permanently conjugated PEG chain to simultaneously improve half-life and disrupt rhIL2 binding to IL2Rα, which suppresses T_reg_ activation while stimulating CD8+ T-cells and NK cells in the tumor microenvironment [284]. Likewise, TransCon IL-2 β/γ consists of a short, permanent PEG chain to eliminate IL2Rα binding coupled with a transiently conjugated 40 kDa PEG for half-life extension [285]. A second class of IL2-based therapeutics biases the cytokine towards IL2Rα binding; NKTR-358 uses permanent PEG conjugation to selectively induce T_reg_ proliferation without expanding CD8+ and CD4+ T-cells and is undergoing multiple Phase 1 trials for treatment of autoimmune disorders [286]. Despite the preclinical promise of these approaches to bias IL2 binding, it is worth noting that multiple programs that restrict IL2Rα binding have already failed in the clinic, with the discontinuation of NKTR-214 and a return to dose-ranging Phase 1/2 studies for SAR444245 [287,288].

#### 3.4.3. Prodrug–Polymer Conjugates

A rapidly growing class of therapeutics utilizes polymers for the preparation of conditionally activated prodrugs. In this approach, the protein–polymer conjugate is inactive but undergoes biotransformation after administration to liberate its active form. By combining the benefits of polymeric half-life extension with stimulus-responsive activation of the therapeutic, this strategy may help maintain serum concentrations within a certain therapeutic window, mitigate altered distribution or attenuated target access as a result of increased size, or localize the therapeutic effect to specific cellular environments.

**Figure 6:**
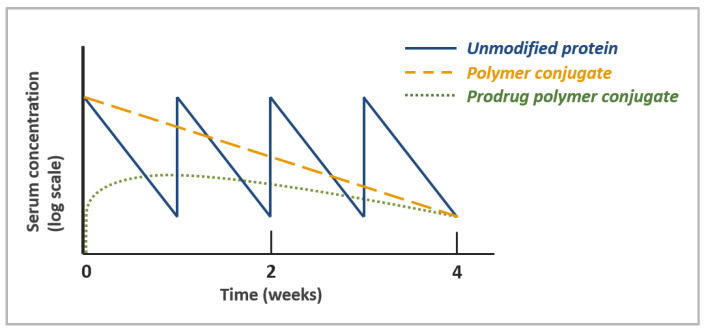
Example PK profiles for an unmodified protein, polymer conjugate with extended systemic half-life, and a prodrug–polymer conjugation that undergoes gradual activation in circulation. First-order clearance is assumed for simplicity.

A subset of clinical polymeric prodrug programs uses stimuli that are not tissue- or target-specific for activation of the protein, such as the transition to physiological pH upon administration. The pharmacokinetic profile of these systems is differentiated from permanent conjugates in that gradual release and activation of the protein leads to more sustained serum concentrations of the active protein at target levels. In contrast, repeated administrations of an unmodified protein leads to rapidly fluctuating serum concentrations, while half-life extension through permanent polymer conjugation is characterized by higher initial C_max_ values and a larger area under the curve (AUC) (Figure 6). The differentiated PK profile of polymeric prodrugs can bring many clinical benefits to programs that would otherwise be limited by their narrow therapeutic window. One such example is Skytrofa, an approved long-acting human growth hormone (hGH) therapy in which the protein is transiently conjugated to a multiarm PEG via a traceless linker. The PEG conjugate is inactive, but the linker slowly hydrolyzes to regenerate native hGH upon exposure to physiological pH. This strategy addresses several shortcomings of previous approaches which used permanent polymer conjugation for half-life extension: (1) gradual activation prevents growth hormone concentrations from reaching supraphysiological levels, which have been associated with deleterious side effects, and (2) release of unmodified GH preserves the distribution pattern of endogenous GH, enabling diffusion across the growth plate [289]. TransCon parathyroid hormone (PTH), which uses a similar approach to maintain physiological PTH concentrations in patients with hypoparathyroidism, recently met its primary endpoint in a Phase 3 clinical trial. If approved, TransCon PTH would be the first long-acting PTH replacement therapy [290,291].

Other polymeric prodrug systems rely on a more localized stimulus for activation. For the treatment of solid tumors, environmental conditions associated with the tumor microenvironment are often used as stimuli, such as low pH, protease activity, or redox activity. For instance, SAR446309 is a T-cell-dependent bispecific antibody (TDB) which incorporates protease-cleavable XTEN masks near each antigen-binding site. Treatment with TDBs is often limited by systemic toxicity such as cytokine-release syndrome; SAR446309 aims to improve the TI of TDB treatment by preventing systemic T-cell activation and localizing activity to the tumor microenvironment, where protease levels are elevated. In nonhuman primates, the maximum tolerated dose of the XTENylated molecule was 400-fold higher than that of the unmasked protein [292]. In the research space, Zhao et al. harnessed nonspecific conjugation to surface-exposed lysines to mask the activity of a range of therapeutic proteins via redox- or acid-responsive PEG chains [293]. They further demonstrated that anti-4-1BB antibody and IL-15 superagonist prodrugs, masked with traceless, reduction-cleavable PEGs, maintained antitumor efficacy while reducing toxicity. As lysine conjugation handles are abundant on the surfaces of most therapeutic proteins, this approach offers a potential generalized strategy for conditional activation of therapeutic proteins in the tumor microenvironment.

Incorporating stimulus-responsive behavior into protein–polymer conjugates may also enable better penetration into tissue targets with low permeability to large macromolecules. For instance, the addition of an MMP-cleavable linker in an INFα-ELP fusion protein improved the tumor penetration of IFNα relative to the bulkier, noncleavable control [294]. Conversely, polymers loaded with both cytotoxic and cell-penetrating peptides were engineered to undergo pH-triggered self-assembly in the tumor microenvironment. This strategy enabled individual polymer conjugates to penetrate more deeply into tumors prior to self-assembling into nanoparticles for enhanced cellular uptake and cytotoxicity [295].

#### 3.4.4. Multivalent Display

One of the most unique and underutilized features of polymer conjugates is their potential for multivalent display, with tailored features such as specific API density, API spacing, and backbone flexibility. This enables a range of properties such as increased potency, enhanced selectivity, and multispecific targeting. Multivalency is fundamental to many biological processes, including the clustering and activation of receptors on cell surfaces [296], viral entry into hosts [297], and the formation of biomolecular condensates to organize a variety of cellular functions [298]. The strength of multivalent interactions is driven by both the enthalpy of binding of the API to its receptor and the combinatorial entropy of the conjugate. Multivalent systems are typically characterized by very slow dissociation kinetics, and the resulting increased avidity enables the development of high-affinity therapeutics from peptides and proteins with low monovalent affinity to their receptors. Moreover, flexible and semiflexible multivalent polymer conjugates permit a greater range of potential binding configurations that lack some of the steric constraints of more rigid systems such as nanoparticles.

The strength of a multivalent interaction is dependent on the ligand spacing, valency, and backbone flexibility. The architectural diversity of polymers, ranging from flexible PEG multimers to dense dendrimers and bottle-brush polymers (Figure 4B), positions them as ideal scaffolds for tuning these multivalent interactions. For instance, conjugation to multiarm PEG polymers significantly increased the affinity of anti-Tie2 agonist antibodies from low micromolar affinity in the anti-Tie2 Fab to activity in the picomolar range for a hexameric PEG-Fab conjugate. Higher valency and higher density, altered by the length and number of PEG arms, led to increased in vitro activity in cellular assays. Treatment with the anti-Tie2 hexamer resulted in a bell-shaped activity curve, suggesting that clustering of the cell-surface Tie2 receptor drives downstream signaling [299].

Multivalent binding has been reported to trigger internalization of internalization-resistant cell-surface receptors through crosslinking-mediated endocytosis [300]. In addition, receptor crosslinking may augment lysosomal trafficking for internalizing receptors [301,302]. In one example, multivalent conjugation of anti-HER2 affibody peptides onto *N-*(2-hydroxylpropyl)methacrylamide (HPMA) induced HER2 crosslinking and triggered receptor internalization [303]. The same group further reported that crosslinking PD-L1 on tumor cells with multivalent PD-L1 antagonist peptide–HPMA conjugates triggered the lysosomal degradation of PD-L1, outperforming an anti-PD-L1 antibody in its ability to prevent tumor relapse after administration of chemotherapy [304].

The ability to incorporate multiple APIs on a single polymer backbone facilitates the delivery of precisely tuned molar ratios of each therapeutic. In addition, loading multiple APIs can unlock unique activity profiles through the synergistic effects of their codelivery; in nature, costimulation via multiple cell-surface receptors forms the basis for many immune system activation pathways. To mimic this behavior, synthetic dendritic cells (sDCs) were produced by conjugating several copies of anti-CD28 and anti-CD3 antibodies to a poly(isocyano peptide) polymer backbone. The inclusion of both antibodies on a single polymer elicited more potent T-cell activation when compared against a mixture of polymers separately conjugated to anti-CD28 and anti-CD3. Furthermore, colocalization of anti-CD28 and anti-CD3 on a single polymer shaped the T-cell response toward the activation of CD8+ effector T-cells and CD4+ helper T-cells while preventing immunosuppressive T_Reg_ activation. The poly(isocyano peptide)-based sDCs also outperformed rigid PLGA microparticles displaying anti-CD3 and anti-CD28 antibodies, an effect that was attributed to the ability of the semiflexible polymer conjugate to more closely mimic the mobility of proteins displayed on a dendritic cell membrane [305].

Finally, multivalent display may give rise to “superselectivity”, in which binding increases nonlinearly with increased receptor density [306,307,308,309]. An optimal superselective system combines high valency with weak monovalent affinity to achieve selectivity to targets with high receptor density. This phenomenon may minimize binding to off-target cell types with lower receptor expression, an intriguing prospect for improving the TI when treating overexpressing cell types such as cancer cells. However, the applications of superselectivity remain largely theoretical, and the ability of these concepts to translate to a clinical setting remains to be seen.

#### 3.4.5. Next-Generation Antibody–Drug Conjugates

Polymers are gaining increasing attention for their ability to improve the biophysical properties of ADCs. High drug loading in conventional ADCs is often limited by the hydrophobicity of the payload, which leads to (1) unfavorable CMC properties, such as poor DP solubility and stability, and (2) poor biological properties, such as rapid clearance and off-target, antigen-independent toxicity. The inclusion of hydrophilic polymers in the ADC linker may mask the hydrophobicity of the drug, leading to more favorable physicochemical properties in the resulting conjugate [310]. The use of polymers in the preparation of ADCs also makes it possible to load multiple drugs onto a single antibody conjugation site. Not only does this increase the maximum feasible DAR for conventional ADCs, potentially enabling the delivery of less potent payloads, but it facilitates the use of novel antibody formats such as nanobodies as well, which may otherwise be limited in their ability to load cargo due to their small size.

Conventional ADCs have been limited by their fast clearance; an effect that has been attributed to the hydrophobicity of the payload. Leon et al. demonstrated that the addition of short PEGs, branched off from the main chain, can effectively mask this hydrophobicity, restore PK, and improve tolerability of a DAR8 MMAE-based ADC [311]. In a subsequent study with nontargeting ADCs, PEG lengths greater than eight units markedly improved survival and reduced neutropenia, and free MMAE concentrations were higher in the groups dosed with unmasked ADC. This led the authors to hypothesize that nonspecific uptake of the hydrophobic ADC led to catabolism, free drug release, and subsequent dose-limiting toxicity [310]. Using a similar rationale, Mablink incorporated short polysarcosine masking groups into the linker of a DAR8 trastuzumab-MMAE ADC and found that a 12-residue sarcosine polymer had superior PK and antitumor efficacy compared to both the PEGylated and unmasked controls [312]. Beyond rescuing PK, increased hydrophilicity in the linker may also increase the physical stability of the ADC during manufacturing and storage, as Buecheler et al. demonstrated a correlation between logP of the payload and ADC aggregation rates upon accelerated stability testing at 40 °C [313].

Mersana’s dolaflexin platform uses hydrophilic polyacetal-based polymers to produce high-DAR ADCs; this technology was used to produce trastuzumab ADCs with DARs up to 20 [36,314]. Multiple dolaflexin-based ADCs are currently in clinical trials. The most advanced program is upfitamab rilsodotin, an anti-NaPi2b antibody loaded with 10–15 auristatin F-hydroxypropylamide payload molecules that is being evaluated for treatment of ovarian cancer [315]. Earlier stage efforts include ASN004, which uses the dolaflexin platform to load payload onto an ScFv-Fc antibody, and dextramabs, which use dextran as a hydrophilic polymer scaffold to load multiple drugs without compromising solubility [316,317]. Finally, while the dolaflexin and dextramab technologies produce heterogeneous ADCs, the use of cysteine-functionalized XTEN polypeptides as a scaffold facilitates the production of homogenous ADCs with DARs as high as 18 [214].

### 3.5. Summary and Remaining Challenges for Polymer Conjugates

The increase in CMC complexity remains a significant barrier in the development of polymer–protein and polymer–peptide conjugates. The design of polymer–protein conjugates, including conjugation site and polymer selection to minimize the loss of activity and maximize stability, is generally an empirical and iterative process. The heterogeneity of the drug product, which stems from many potential factors, including the polydispersity of the polymer, heterogeneous conjugation to multiple sites in the protein, or gradual deconjugation, complicates the control system for polymer conjugates once they enter development. Similarly, the need for additional purification steps and novel analytical characterization techniques compared to traditional large molecules requires manufacturers to deviate from platform processes and assays. The viscosity of a polymer conjugate is often higher than that of the unmodified protein as well; in high-dose products, viscosity at the target DP concentration can exceed limits for TFF or injection, requiring the development of novel manufacturing processes and specialized autoinjectors. All of these factors tend to drive up manufacturing costs and timelines.

Many recent developments offer novel strategies to reduce the manufacturing complexity of these programs; site-specific conjugation chemistries permit the selection of conjugation sites that are more distal to the antigen-binding site, while grafting from approaches reduce the complexity of purification process design. Meanwhile, genetic fusion of disordered polypeptides eliminates the need for separate conjugation processes entirely and produces homogeneous conjugates, although they are limited by their narrow range of conjugation sites and architectures. Other approaches aim to improve drug product stability through polymer conjugation, with the goal of enabling longer shelf lives, aqueous formulations, or room-temperature storage, which each add a layer of convenience for patients and healthcare professionals. In addition, molecular dynamics simulations of polymer–protein conjugate systems have revealed new insights into the interactions between polymers and proteins and their impact on stability [318,319].

**Table 6:** Polymer–protein and polymer–peptide conjugates in the clinic.

Name	Polymer	API	Route of Administration	Phase	Proposed Mechanism	References
Pegcetacoplan	PEG	Peptide-based C3 inhibitor (compstatin derivative)	ITV	NDA	PEGylation extends vitreous half-life	[320,321,322]
Pegunigalsidase alfa	PEG	α-galactosidase-A	IV	BLA	Dimerization of the enzyme with a homobifunctional PEG and additional surface PEGylation improves systemic half-life and reduces immunogenicity	[323,324]
Daprolizumab pegol	PEG	Anti-CD40L Fab	IV	3	PEGylation improves systemic half-life and mitigates potential for Fc-mediated platelet crosslinking	[323,324,325]
BIVV001	XTEN	Factor VIII	IV	3	Steric shielding from XTEN, in combination with FcRn recycling from Fc fusion, improves FVIII half-life	[326]
KSI-301	Phosphoryl- choline	aVEGF	ITV	3	Polymer conjugation extends vitreous half-life	[275]
Transcon PTH	PEG	Parathyroid hormone (1-34)	SC	3	Cleavable PEG masks activity and maintains PTH concentrations within normal physiological levels	[290]
Upifitamab Rilsodotin	Polyacetal	NaPi2b conjugated to auristatin derivative	IV	3	DAR10-15 ADC using Mersana Dolaflexin platform	[36,290]
Pegargiminase	PEG	Arginine deiminase	IM	3	PEGylation improves systemic half-life and reduces immunogenicity	[327]
Transcon CNP	PEG	C-type natriuretic peptide	SC	2	Cleavable PEGylation extends the dosing frequency from daily to weekly and may reduce C_max_-driven adverse events	[328,329]
Sanguinate	PEG	Bovine hemoglobin	IV	2	PEGylation reduces extravasation and immunogenicity	[330]
NKTR-358	PEG	IL-2	IV	2	Permanent PEG conjugation biases IL-2 towards T_reg_ activation for treatment of autoimmune disease	[286,331]
NKTR-255	PEG	IL-15	IV	2	PEGylation improves systemic half-life	[332]
SAR444245	PEG	IL-2	IV	1/2	Permanent PEG conjugation in the IL-2Rα binding site suppresses T_reg_ activation	[287,333]
Pegozafermin	PEG	FGF21	SC	2	PEGylation improves systemic half-life	[334]
TransCon IL-2 β/γ	PEG	IL-2	IV	1	Permanent PEG conjugation in the IL-2Rα binding site suppresses T_reg_ activation; transient conjugation to a second 40 kDa PEG extends half-life and reduces C_max_	[285]
SAR446309	XTEN	aHER2/CD3 bispecific	IV	1	Protease-cleavable XTEN masks activity and improves TI	[292]
ASN004	Polyacetal	5T4	IV	1	DAR10-12 ScFv-Fc ADC using Mersana Dolaflexin platform	[316]
AZD8205	PEG_8_	B7-H4	IV	1	Inclusion of PEG in the ADC linker improves serum stability and increases the therapeutic window	[335]

The COVID-19 pandemic has prompted an increased industry focus on patient-centricity, and polymer conjugates are well-positioned to have a continued impact in this space. Many parenterally administered polymer–protein conjugates have been proven to enhance the treatment experience and improve outcomes for patients through reduced dosing frequency. Polymer conjugates are anticipated to be similarly impactful in ophthalmology in the coming years. Multiple late-stage clinical programs use polymer conjugation for half-life extension in the vitreous, potentially allowing a more convenient dosing regimen and improved visual acuity outcomes for patients by ensuring that they receive the required drug exposure long-term. Beyond reduced dosing frequency, polymeric prodrugs have already shown their potential to maintain serum concentrations within the optimal therapeutic window; this approach offers great potential for reducing C_max_-driven toxicity and preserving the tissue distribution of the unmodified peptide or protein. The combined impact of both improved safety and reduced dosing frequency offered by polymer conjugation may allow some of these treatments to transition to the home administration setting, reducing the overall burden on healthcare systems and providing a more convenient option for patients.

Several emerging classes of polymer conjugates reach beyond the modulation of pharmacokinetics as their mechanisms of action. For example, tissue-specific polymer prodrugs offer a more targeted approach to improve the TI of a protein by preferentially activating the protein when it reaches the target tissue. Similarly, the use of polymers to bias the selectivity of endogenous proteins has garnered excitement in the immunology space; these programs may enable researchers to better harness the power of potent but pleiotropic immune modulators such as cytokines. Finally, the inclusion of polymers in ADCs may permit higher drug loading without sacrificing PK, enabling the use of less potent drugs or the delivery of larger quantities of drug to cells with lower target-expression levels.

Other concepts, such as multivalent display of one or multiple ligands, have shown great promise in the preclinical space for their ability to confer profoundly different biological activity in the resulting conjugate. However, the increased CMC complexity of these systems may be partially responsible for their limited clinical use to date; for example, the cumulative effect of the stochastic conjugation of the therapeutic and the polydispersity of the polymer backbone significantly increases the heterogeneity of a multivalent conjugate. Continued advances in the synthesis of low-dispersity or chemically defined polymers, combined with improved in vitro analytical characterization and screening techniques, may facilitate the translation of some of these more complex systems into clinical programs.

## 4. Conclusions and Outlook

In this review, we highlighted the remarkable versatility of proteins and peptides in the development of chemically conjugated therapeutics, demonstrating their ability to function as either the API itself or as the delivery vehicle. Conjugation is of great interest because it can be used not only to improve the existing drug product properties but also to impart entirely new properties. As a result, chemically modified proteins or chemicals enhanced by protein conjugation can have far-reaching effects, such as modulation of PK, improvement in safety and tolerability, or entry into difficult-to-access compartments, including BBB transcytosis and intracellular delivery. In addition, the impact of chemically modified protein/peptide medicines on therapeutic areas is vast, with applications in neurology, immunology, oncology, ophthalmology, muscle disorders, and endocrine disorders. The abundance of clinical- and commercial-stage protein and peptide conjugates across these disease areas speaks to the merit of these subclasses of chemically enhanced therapeutics.

A common challenge shared by all formats discussed in this review is their complexity; the need to simultaneously optimize the protein/peptide, linker, and polymer/payload complicates the discovery campaign, manufacturing, and development of a control system for protein and peptide conjugates. While the current clinical success of these conjugates is attributed to the successful collaboration between biologists and synthetic chemists, the therapeutic potential of next-generation conjugates may only be fully realized by further interfacing with automation engineers and computational chemists to design in silico predictive tools and high-throughput screening (HTS) techniques. Much of the success of traditional monoclonal antibodies can be ascribed to the development of high-throughput assays to guide candidate selection, ranging from in silico tools to predict developability through in vitro display technologies to screen billions of candidates for target-binding. Meanwhile, many of these tools are still lacking for protein conjugate systems.

Further computational studies will likely strengthen the field’s mechanistic understanding of the interactions of proteins with their various partners and the resulting impact on physicochemical properties such as biological activity and stability. In addition, the development of high-throughput in vitro and in vivo screening tools may allow researchers to sample a wider design space and more rapidly drive toward favorable conjugate properties. For instance, the polymer conjugate field would benefit greatly from the synthesis of polymer libraries to systematically explore the impact of polymer properties on stability, viscosity, and antigen binding. Similarly, for ADCs, predictive tools for the structure–activity relationship capable of examining all possible combinations of the various modular components would be a boon to the field, where much of our current understanding of molecule design comes from clinical observations late in the development pipeline. Collectively, insights from modeling and HTS can then be used to guide a more rational design process for future protein conjugates, enabling them to continue to play a major role in the development of next-generation therapeutics.

## Figures and Tables

**Table 1:** FDA-approved ADCs.

ADC	Target	Antibody Isotype	Conjugation Site	Linker	Payload/ Payload Class	Payload MOA	DAR	Disease Indication (Approval Year)
Mylotarg^®^ (gemtuzumab ozogamicin)	CD33	IgG4	Lysines	acid cleavable	ozogamicin/ calicheamicin	DNA damaging	2–3	Acute myeloid leukemia (2000) withdrawn 2010, reapproved 2017 with alternative dosing regimen
Adcetris^®^ (brentuximab vedotin)	CD30	IgG1	Interchain disulfides	enzyme cleavable	MMAE/ auristatin	microtubule inhibitor	4	Hodgkin lymphoma, anaplastic large cell lymphoma, CD30+ mycosis fungoides (2011)
Kadcyla^®^ (trastuzumab emtansine)	HER2	IgG1	Lysines	noncleavable	DM1/ maytansinoid	microtubule inhibitor	3.5	HER2+ metastatic breast cancer (2013)
Besponsa^®^ (inotozumab ozogamicin)	CD22	IgG4	Lysines	acid cleavable	ozogamicin/ calicheamicin	DNA damaging	6	B-cell acute lymphoblastic leukemia (2017)
Polivy^®^ (polatuzumab vedotin)	CD79b	IgG1	Interchain disulfides	enzyme cleavable	MMAE/ auristatin	microtubule inhibitor	3.5	Diffuse large B-cell lymphoma (2019)
Padcev^®^ (enfortumab vedotin)	Nectin-4	IgG1	Interchain disulfides	enzyme cleavable	MMAE/ auristatin	microtubule inhibitor	3.8	Locally advanced or metastatic urothelial cancer (2019)
Enhertu^®^ (trastuzumab deruxtecan)	HER2	IgG1	Interchain disulfides	enzyme cleavable	DXd/ camptothecin	TOP1 inhibitor	8	HER2+ unresectable or metastatic breast cancer (2021)
Trodelvy^®^ (sacituzumab govitecan)	TROP2	IgG1	Interchain disulfides	acid cleavable	SN-38/ camptothecin	TOP1 inhibitor	7.6	Locally advanced or metastatic HER2+ breast cancer (2019)
Blenrep^®^ (belantamab mafodotin)	BMCA	IgG1 (a-fucosylated)	Interchain disulfides	noncleavable	MMAF/ auristatin	microtubule inhibitor	4	Multiple myeloma (2020) *withdrawn 2022*
Zynlonta^®^ (loncastuximab tesirine)	CD19	IgG1	Interchain disulfides	enzyme cleavable	SG3199/PBD dimer	DNA damaging	2.3	Large B-cell lymphoma (2021)
Tivdak^®^ (tisotumab vedotin)	Tissue Factor	IgG1	Interchain disulfides	enzyme cleavable	MMAE/ auristatin	microtubule inhibitor	4	Recurrent or metastatic cervical cancer (2021)
Elahere^®^ (mirvetuximab soravtansine)	Folate receptor alpha	IgG1	Lysines	Reducible disulfide	DM4/ maytansinoid	microtubule inhibitor	3–4	Platinum-resistant epithelial ovarian, fallopian tube, or primary peritoneal cancer (2022)

BCMA: B-cell maturation antigen; DM1: derivative of maytansine 1 [N2′-deacetyl-N2′-(3-mercapto-1-oxopropyl)-maytansine]; DM4: derivative of maytansine 4 [N2′-deacetyl-N2′-(4-mercapto-4-methyl-1-oxopentyl) maytansine] DM4: maytansine derivative; Dxd: exatecan derivative; HER2: human epidermal growth factor receptor 2; MOA: mechanism of action; TOP1: topoisomerase I; TROP2: tumor-associated calcium signal transducer 2; SN-38: active metabolite of the topoisomerase I inhibitor irinotecan.

**Table 3:** Peptide–oligonucleotide conjugates with published preclinical or in vitro data.

Oligo Type	Indication	Peptide	Formulation Type	Target	Current Stage	Reference
ASO	Histiocytic lymphoma cell	Protamine	Noncovalent nanosuspension	c-myc	in vitro	[88]
ASO	HIV-AIDS	Protamine	Covalent conjugate/nanoparticle	HIV-1	in vitro	[89]
PMO	Cell proliferation disorders	Arginine-rich CPP	Covalent conjugate	c-myc	preclinical	[90]
PMO	N/A	Bicyclic CPP	Covalent conjugate	mutant intron from the human β-globin gene	in vitro	[91]
PMO	N/A	Tat CPP	Covalent conjugate	mutant splice site of the human globin β-thalassemic intron 2	in vitro	[92]
PMO SSO	DMD	B-MSP	Peptide fusion-conjugate	heart and skeletal muscle	preclinical	[93]
PMO SSO	DMD	Arginine-rich CPP	Covalent conjugate	muscle	preclinical	[94]
PMO SSO	SMA	Pip6a	Covalent conjugate	ISS-N1	preclinical	[81]
PNA	HIV	Transportan/ R6-Penetratin	Covalent conjugate	HIV-1+ HeLa cells	in vitro	[95]
siRNA	HIV	Protamine-Fab fusion	Noncovalent conjugate	HIV-1	preclinical	[96]
siRNA	Melanoma	Protamine-ScFv fusion	Noncovalent conjugate	ErbB2	preclinical	[96]
siRNA	Neurodegeneration	Penetratin1	Covalent conjugate	SOD1/Casp3 in neurons	in vitro	[97]
siRNA	Colon cancer	LMWP	Covalent conjugate	eGFP	in vitro	[98]
siRNA	HIV	Protamine-ScFv fusion	Noncovalent conjugate	Ku70	preclinical	[99]

B-peptide: arginine-rich cell-penetrating peptide; LMWP: low molecular weight protamine; MSP: muscle targeting heptapeptide; Pip6a: peptide internalization; PNA: peptide nucleic acid; ScFv: single-chain variable fragment; SSO: splice-switching oligonucleotide; Tat: transactivator of transcription from HIV-1; R6: hexa arginine; X = 6-aminohexanoic acid.

**Table 4:** Approved pneumococcal conjugate vaccines (PCV).

Name	Commercial Product	Serotypes	Carrier Protein	Year
PPV23	Pneumovax^®^23	1, 2, 3, 4, 5, 6B, 7F, 8, 9N, 9V, 10A, 11A, 12F, 14, 15B, 17F, 18C, 19A, 19F, 20, 22F, 23F, and 33F	None	1983
PCV7	Prevnar^®^	4, 6B, 9V, 14, 18C, 19F, and 23F	Attenuated diphtheria protein CRM197	2000
PCV10	Synflorix™	1, 4, 5, 6B, 7F, 9V, 14, 18C, 19F, and 23F	NTHi, diphtheria toxoid, tetanus toxoid	2009
PCV13	Prevnar13^®^	1, 3, 4, 5, 6A, 6B, 7F, 9V, 14, 19A, 19F, 18C, and 23F	Attenuated diphtheria protein CRM197	2010
PCV15	Vaxneuvance™	1, 3, 4, 5, 6A, 6B, 7F, 9V, 14, 18C, 19A, 19F, 22F, 23F, and 33F	Attenuated diphtheria protein CRM197	2021
PCV20	Prevnar20™	1, 3, 4, 5, 6A, 6B, 7F, 8, 9V, 10A, 11A, 12F, 14, 15B, 18C, 19A, 19F, 22F, 23F, and 33F	Attenuated diphtheria protein CRM197	2021

**Table 5:** Approved polymer–protein and polymer–peptide conjugates.

Name	Polymer	API	Route of Administration	Indication	FDA Approval Year
Stimufend^®^ (pegfilgrastim-fpgk)	PEG	Recombinant human G-CSF	SC	Febrile neutropenia	2022
Rolvedon^®^ (eflapegrastim-xnst)	PEG	Recombinant human G-CSF	SC	Febrile neutropenia	2022
Besremi^®^ (ropeginterferon alfa-2b-njft)	PEG	Recombinant interferon alfa-2b	SC	Polycythemia vera	2021
Skytrofa^®^ (lonapegsomatropin-tcgd)	PEG	Somatropin	SC	Pediatric growth hormone deficiency	2021
Empaveli^®^ (pegcetacoplan)	PEG	Peptide-based C3 inhibitor (compstatin derivative)	SC	Paroxysmal nocturnal hemoglobinuria	2021
Fylnetra^®^ (pegfilgrastim-pbbk)	PEG	Recombinant human G-CSF	SC	Febrile neutropenia	2021
Nyvepria^®^ (pegfilgrastim-apgf)	PEG	Recombinant human G-CSF	SC	Febrile neutropenia	2020
Ziextenzo^®^ (pegfilgrastim-bmez)	PEG	Recombinant human G-CSF	SC	Febrile neutropenia	2019
Esperoct^®^ (antihemophilic factor (recombinant), glycopegylated-exei)	PEG	Recombinant factor VIII	IV	Hemophilia A	2019
Asparlas^®^ (calaspargase pegol-mknl)	PEG	L-asparaginase	IV	Acute lymphoblastic leukemia	2018
Udenyca^®^ (pegfilgrastim-cbqv)	PEG	Recombinant human G-CSF	SC	Febrile neutropenia	2018
Revcovi^®^ (elapegademase-lvlr)	PEG	Recombinant adenosine deaminase	IM	Adenosine deaminase severe combined immune deficiency	2018
Jivi^®^ (antihemophilic factor (recombinant), PEGylated-aucl)	PEG	Recombinant factor VII	IV	Hemophilia A	2018
Fulphila^®^ (pegfilgrastim-jmdb)	PEG	Recombinant human G-CSF	SC	Febrile neutropenia	2018
Palynziq^®^ (pegvaliase-pqpz)	PEG	Recombinant phenylalanine ammonia lyase	SC	Phenylketonuria	2018
Rebinyn^®^ (Jivi^®^ (antihemophilic factor (recombinant), PEGylated-aucl)	PEG	Recombinant factor IX	IV	Hemophilia B	2017
Adynovate^®^ (rurioctocog alfa pegol)	PEG	Recombinant factor VIII	IV	Hemophilia A	2015
Plegridy^®^ (peginterferon beta-1a)	PEG	Recombinant interferon beta-1a	SC, IM	Multiple sclerosis	2014
Omontys^®^ (peginesatide)	PEG	Peptide-based erythropoietin receptor agonist	SC, IV	Anemia associated with chronic kidney disease	2012 (Withdrawn 2019)
Sylatron^®^ (peginterferon alfa-2b	PEG	Recombinant interferon alfa-2b	SC	Melanoma	2011
Krystexxa^®^ (pegloticase)	PEG	Recombinant uricase	IV	Gout	2010
Cimzia^®^ (certolizumab pegol)	PEG	Anti-TNF-ɑ Fab′	SC	Crohn’s disease	2008
Mircera^®^ (methoxy polyethylene glycol-epoetin beta)	PEG	Recombinant erythropoietin	IV, SC	Anemia associated with chronic kidney disease	2007
Somavert^®^ (pegvisomant)	PEG	Human growth hormone analog, growth hormone receptor antagonist	SC	Acromegaly	2003
Neulasta^®^ (pegfilgrastim)	PEG	Recombinant human G-CSF	SC	Febrile neutropenia	2002
Pegasys^®^ (peginterferon alfa-2a)	PEG	Recombinant interferon alfa-2a	SC	Chronic hepatitis B and C	2002
Pegintron ^®^	PEG	Recombinant interferon alfa-2b	SC	Chronic hepatitis C, melanoma	2001
Oncaspar^®^ (pegaspargase)	PEG	L-asparaginase	IM, IV	Acute lymphoblastic leukemia	1994
Adagen^®^ (pegademase bovine)	PEG	Bovine adenosine deaminase	IM	Adenosine deaminase severe combined immune deficiency	1990

## Data Availability

Not applicable.

## Abbreviations

3p-hpRNA	5′ Triphosphate hairpin RNA
ABC	Accelerated blood clearance
AD	Adenosine deaminase
ADA	Antidrug antibodies
ADC	Antibody–drug conjugate
ADCC	Antibody-dependent cell-mediated cytotoxicity
ADCP	Antibody-dependent cell-mediated phagocytosis
AOC	Antibody–oligonucleotide conjugate
APA	Anti-PEG antibodies
ASO	Antisense oligonucleotide
ATRP	Atom-transfer radical polymerization
AUC	Area under the curve
BBB	Blood–brain barrier
BCMA	B-cell maturation antigen
bis-MPA	Poly-2,2-bis(hydroxymethyl)propionic acid
BLA	Biologics license application
B-peptide	Arginine-rich cell-penetrating peptide
BSA	Bovine serum albumin
CDC	Complement dependent cytotoxicity
CMC	Chemistry, manufacturing, and controls
CMV	Cytomegalovirus
CNS	Central nervous system
CpG	Cytosine–phosphate–guanine dinucleotide
CPP	Cell-penetrating peptide
CRM_197_	Attenuated form of *C. diphtheriae* toxin
DAR	Drug to antibody ratio
DBCO	Dibenzocyclooctyne
DP	Drug product
DM1*	Myotonic dystrophy type 1
DS	Drug substance
DT	Diphtheria toxoid
DVD	Dual variable domain antibody
dsASO	Double-stranded ASO
EGFR	Epidermal growth factor receptor
ELP	Elastin-like polypeptides
Fab	Antibody fragment
Fc	Antibody constant fragment
FcRn	Neonatal Fc receptor
FDA	Food and Drug Administration
FSHD	Facioscapulohumeral muscular dystrophy
GA	Geographic atrophy
GAC	*Streptococcus pyrogen* group A carbohydrate
GalNac	N-Acetylgalactosamine
G-CSF	Human granulocyte colony-stimulating factor
GFP	Green Fluorescent Protein
GMAB	Modified lupus autoantibody
HA	Hyaluronic acid
hGH	Human growth hormone
HiB	*Haemophilus influenzae serotype b*
HIV-1	Human immunodeficiency virus type I
HPMA	(2-hydroxylpropyl)methacrylamide
HTS	High-throughput screening
IM	Intramuscular
IND	Investigational new drug
IP	Intraperitoneal
IT	Intrathecal
ITV	Intravitreal
IV	Intravenous
LMWP	Low molecular weight protamine
LNP	Lipid nanoparticle
mAb	Monoclonal antibody
MG	Myasthenia gravis
miR	MicroRNA
MMAE	Monomethyl auristatin E
MMAF	Monomethyl auristatin F
MMC	Multiple myeloma cells
MSP	Muscle targeting heptapeptide
nAMD	Neovascular age-related macular degeneration
N/A	Not available
NDA	New drug application
NHS	N-hydroxysuccinimide
nnAA	Non-native amino acid
NTHi	*Haemophilus influenzae*
OPS	O antigen polysaccharides
OMPC	Outer membrane protein complex of serogroup B meningococcus
PABC	Para-amino benzyloxycarbonyl
PAD	Peripheral artery disease
pAMF	P-azidomethyl phenylalanine
PAMAM	Poly(amidoamine)
PAS	Proline/alanine-rich sequences
PBD	Pyrrolobenzodiazepine
pCB	Poly(carboxybetaine)
PCV	Pneumococcal conjugate vaccine
PD	*Haemophilus influenzae* protein D
PEG	Polyethylene glycol
PK	Pharmacokinetics
PMO	Phosphorodiamidate morpholino oligonucleotide
PNA	Peptide nucleic acid
PNAG	Poly-N-acetylglucosamine
POEGMA	Poly(oligo(ethylene glycol) methyl ether methacrylate
POZ	Poly(2-oxazoline)
PPC	Polysaccharide-based vaccine
pPC	Poly(phosphorylcholine)
PROTAC	Proteolysis targeting chimera
PRP	Polyribosyl ribitol phosphate
PSA	Polysialic acid
pSar	Polysarcosine
pSB	Poly(sulfobetaine)
PTH	Parathyroid hormone
pTMAO	Poly(trimethylamine N-oxide
RAFT	Reversible addition-fragmentation chain transfer polymerization
*r*EPA	*Pseudomonas aeruginosa* protein exotoxin A
RNAi	RNA inhibition
RVO	Retinal vein occlusion
SC	Subcutaneous
ScFv	Single-chain variable fragment
sDC	Synthetic dendritic cell
siRNA	Small interfering RNA
SLO	Streptolysin O
SMA	Spinal muscular atrophy
SSO	Splice-switching oligonucleotide
TAT	Transactivator of transcription from HIV-1
TDB	T-cell-dependent bispecific antibody
TFF	Tangential flow filtration
TfR1	Transferrin receptor
TI	Therapeutic index
TLR	Toll-like receptor
T_m_	Melting temperature
TNF	Tumor necrosis factor
TT	Tetanus toxoid
Un-dPP	undecaprenyl pyrophosphate
Val-Cit	Valine-citrulline
VEGF	Vascular endothelial growth factor
VLP	Virus-like particle/nanoparticle
WHO	World Health Organization
